# Diclofenac and other non-steroidal anti-inflammatory drugs (NSAIDs) are competitive antagonists of the human P2X3 receptor

**DOI:** 10.3389/fphar.2023.1120360

**Published:** 2023-03-16

**Authors:** Laura Grohs, Linhan Cheng, Saskia Cönen, Bassam G. Haddad, Astrid Bülow, Idil Toklucu, Lisa Ernst, Jannis Körner, Günther Schmalzing, Angelika Lampert, Jan-Philipp Machtens, Ralf Hausmann

**Affiliations:** ^1^ Institute of Clinical Pharmacology, RWTH Aachen University, Aachen, Germany; ^2^ Department of Neurology, University Hospital, RWTH Aachen University, Aachen, Germany; ^3^ Molecular and Cellular Physiology (IBI-1), Institute of Biological Information Processing (IBI), Forschungszentrum Jülich, Jülich, Germany; ^4^ Department of Plastic Surgery, Hand Surgery—Burn Center, University Hospital, RWTH Aachen University, Aachen, Germany; ^5^ Institute of Physiology (Neurophysiology), RWTH Aachen University, Aachen, Germany; ^6^ Institute for Laboratory Animal Science and Experimental Surgery, RWTH Aachen University, Aachen, Germany; ^7^ Department of Anesthesiology, University Hospital, RWTH Aachen University, Aachen, Germany

**Keywords:** P2X3 receptor, nociception, chronic pain, non-steroidal anti-inflammatory drugs (NSAIDs), competitive antagonist, drug screening

## Abstract

**Introduction:** The P2X3 receptor (P2X3R), an ATP-gated non-selective cation channel of the P2X receptor family, is expressed in sensory neurons and involved in nociception. P2X3R inhibition was shown to reduce chronic and neuropathic pain. In a previous screening of 2000 approved drugs, natural products, and bioactive substances, various non-steroidal anti-inflammatory drugs (NSAIDs) were found to inhibit P2X3R-mediated currents.

**Methods:** To investigate whether the inhibition of P2X receptors contributes to the analgesic effect of NSAIDs, we characterized the potency and selectivity of various NSAIDs at P2X3R and other P2XR subtypes using two-electrode voltage clamp electrophysiology.

**Results:** We identified diclofenac as a hP2X3R and hP2X2/3R antagonist with micromolar potency (with IC_50_ values of 138.2 and 76.7 µM, respectively). A weaker inhibition of hP2X1R, hP2X4R, and hP2X7R by diclofenac was determined. Flufenamic acid (FFA) inhibited hP2X3R, rP2X3R, and hP2X7R (IC_50_ values of 221 µM, 264.1 µM, and ∼900 µM, respectively), calling into question its use as a non-selective ion channel blocker, when P2XR-mediated currents are under study. Inhibition of hP2X3R or hP2X2/3R by diclofenac could be overcome by prolonged ATP application or increasing concentrations of the agonist α,β-meATP, respectively, indicating competition of diclofenac and the agonists. Molecular dynamics simulation showed that diclofenac largely overlaps with ATP bound to the open state of the hP2X3R. Our results suggest a competitive antagonism through which diclofenac, by interacting with residues of the ATP-binding site, left flipper, and dorsal fin domains, inhibits the gating of P2X3R by conformational fixation of the left flipper and dorsal fin domains. In summary, we demonstrate the inhibition of the human P2X3 receptor by various NSAIDs. Diclofenac proved to be the most effective antagonist with a strong inhibition of hP2X3R and hP2X2/3R and a weaker inhibition of hP2X1R, hP2X4R, and hP2X7R.

**Discussion:** Considering their involvement in nociception, inhibition of hP2X3R and hP2X2/3R by micromolar concentrations of diclofenac, which are rarely reached in the therapeutic range, may play a minor role in analgesia compared to the high-potency cyclooxygenase inhibition but may explain the known side effect of taste disturbances caused by diclofenac.

## 1 Introduction

P2X receptors (P2XR) constitute a family of non-selective cation channels gated by extracellular ATP (North and Barnard, 1997). Seven different subtypes (P2X1–7) can assemble into homo- or heterotrimers (Nicke et al., 1998; North, 2002).

The P2X3R, which is expressed in sensory neurons (Chen et al., 1995), plays a crucial role in nociception (Burnstock, 2016). P2X3-deficient mice exhibit an attenuated pain behavior after injection of ATP into the hind paw compared to wild-type mice (Cockayne et al., 2000), whereas the response to acute mechanical pain stimuli remains unchanged (Souslova et al., 2000). Accordingly, pharmacological inhibition of P2X3R has been shown to effectively reduce chronic or neuropathic pain in rodents (Jarvis et al., 2002). Recently, the modulator TMEM163, a 289-amino acid transmembrane protein, was shown to be required for the complete function of the neuronal P2X3R- and P2X4R- and pain-related ATP-evoked behavior in mice (Salm et al., 2020). In addition to P2X3R, involvement in nociception could also be assigned to heterotrimeric P2X2/3R, P2X4R, and P2X7R (Chessell et al., 2005; Cockayne et al., 2005; Tsuda et al., 2009). All of these seem to be more relevant for the development of neuropathic or inflammatory pain than for acute nociception (Chessell et al., 2005; Tsuda et al., 2009).

The significant role of P2X3R in nociception makes P2X3R a promising target for the development of new analgesics (North and Jarvis, 2013). However, until now, none of the developed antagonists has been approved for clinical use as an analgesic, even if gefapixant (formerly AF-219) is approved as an anti-cough agent in Japan (details are given as follows). One of the first potent and selective P2X3R (and P2X2/3R) antagonists was A-317491, which successfully reduced chronic pain in rodent models (Jarvis et al., 2002), but showed insufficient distribution in the central nervous system (Sharp et al., 2006). Several other P2X3R antagonists have been developed as clinical candidates, such as AF-219/gefapixant, BAY-1817080/eliapixant, BLU-5937, MK-3901, or S-600918/sivopixant (Spinaci et al., 2021; Niimi et al., 2022). The availability of the crystal structures of the human P2X3R (Mansoor et al., 2016), together with cryo-EM techniques, is ideally suited to facilitate structure-based drug design for P2X3Rs by revealing and characterizing novel ligand-binding sites (Oken et al., 2022).

The most advanced is the development of gefapixant, a P2X3R and P2X2/3R antagonist, which effectively reduced chronic cough caused by hypersensitivity of the cough reflex in phase 2 and 3 trials (Abdulqawi et al., 2015; Marucci et al., 2019). However, a disturbance in taste sensation was described as a side effect by all patients (Abdulqawi et al., 2015). Gefapixant is a first-in-class, non-narcotic selective P2X3 receptor antagonist and was recently approved for marketing in Japan as a treatment option for refractory or unexplained chronic cough (Markham, 2022). Another promising substance, BLU-5937, was able to effectively reduce chronic cough in animal models without altering taste sensation, possibly due to its considerably higher selectivity for P2X3R *versus* P2X2/3R (Garceau and Chauret, 2019). BLU-5937 is now part of a phase 2 study for the treatment of chronic cough (Marucci et al., 2019). Also, sivopixant was shown to reduce objective cough frequency and improved health-related quality of life, with a low incidence of taste disturbance, among patients with a refractory or unexplained chronic cough in a phase 2a trial (Niimi et al., 2022). Eliapixant showed favorable tolerability with no taste-related adverse events in its first-in-human study, and in a phase 1/2a study, eliapixant administration showed reduced cough frequency and severity and was well-tolerated with acceptable rates of taste-related events (Morice et al., 2014; Klein et al., 2022).

In light of the promising role of P2X3R antagonists for the treatment of pain and refractory cough, as well as the high likelihood of taste disturbances caused by not fully selective P2X3R antagonists (against heteromeric P2X2/3R), it appears interesting to investigate whether already approved drugs do affect the P2X3R-mediated responses. For this purpose, a screening of 2000 approved drugs, natural products, and bioactive substances was performed in a previous study of our group. In this screening, aurintricarboxylic acid (ATA) was identified as a potent P2X1R and P2X3R antagonist (Obrecht et al., 2019). An inhibitory effect could also be demonstrated for other drugs. These included various non-steroidal anti-inflammatory drugs (NSAIDs), and diclofenac showed the highest inhibitory effect of the screened NSAIDs. The analgesic, antipyretic, and anti-inflammatory effect of NSAIDs is generally described to result from the inhibition of prostaglandin synthesis by inhibiting cyclooxygenases COX-1 and COX-2 (Vane, 1971). Most NSAIDs constitute reversible, competitive blockers of the enzyme cyclooxygenase (COX), while acetylsalicylic acid (Aspirin^®^) can cause irreversible inactivation of COX through acetylation of serine 530 (Rome and Lands, 1975; DeWitt et al., 1990).

Considering the involvement of P2X3R in nociception, it is conceivable that inhibition of P2X3R by NSAIDs represents an additional mode of action besides COX inhibition. To investigate whether the inhibition of P2XR contributes to the analgesic effect of NSAIDs, we determined the potency and selectivity of various NSAIDs at P2X3R and other P2XR subtypes using two-electrode voltage clamp (TEVC) electrophysiology. The investigated NSAIDs included diclofenac, ibuprofen, flunixin, meclofenamic acid, naproxen, and flufenamic acid (FFA). The latter was chosen because it additionally plays an important role in research as a non-selective ion channel blocker (Guinamard et al., 2013).

In the present study, we have for the first time shown that diclofenac is a hP2X3R and hP2X2/3R antagonist with micromolar potency. Our results strongly support a competitive antagonism through which diclofenac, by interacting with residues of the ATP-binding site, left flipper, and dorsal fin domains, inhibits the gating of P2X3R by conformational fixation of the left flipper and dorsal fin domains. In addition, a weaker inhibition of hP2X1R, hP2X4R, and hP2X7R was shown. Less potent inhibition of hP2X3R was observed for all other investigated NSAIDs. FFA was proven to significantly inhibit hP2X3R, rP2X3R, and hP2X7R.

## 2 Materials and methods

### 2.1 Chemicals

The investigated NSAIDs and most standard chemicals were purchased from Sigma–Aldrich/Merck (Taufkirchen, Germany), if not otherwise specified. ATP sodium salt and α,β-meATP were purchased from Roche Diagnostics (Mannheim, Germany) and Tocris Bioscience (Bristol, United Kingdom), respectively. Collagenase type 2 was purchased from Worthington Biochemical Corp (Lakewood, United States and distributed by CellSystems, Troisdorf, Germany).

### 2.2 Expression of P2X receptors in *X. laevis* oocytes

Oocyte expression plasmids encoding the wild-type (wt) and N-terminally His-tagged (His-) fusion constructs of the hP2X2R, hP2X3R, hP2X4R, and hP2X7R, and the mutant His-^20^RMVL^23^KVIV^23^S^26^N-hP2X1R, S^15^V-rP2X3R, and His-S^15^V-hP2X3R were the same as used in previous research (Hausmann et al., 2006; Wolf et al., 2011; Hausmann et al., 2014; Obrecht et al., 2019). In most cases, the N-terminal His-tagged variants of P2XRs were used here and in the previous studies to allow biochemical analyses on affinity-purified proteins using the same constructs. Although this was not required for the present study, it allows for better comparability with our previous studies. Capped cRNAs of different P2XRs were already available in the research group or were synthesized as previously described (Schmalzing et al., 1991; Stolz et al., 2015). cRNA was injected into collagenase-defolliculated *X. laevis* oocytes in aliquots of 41 nl or 23 nl (see Supplementary Table S1 for the amount of cRNA used for expression of the indicated P2XR) using a Nanoliter 2000 injector (World Precision Instruments, Sarasota, United States of America) as described previously (Hausmann et al., 2014; Stolz et al., 2015; Obrecht et al., 2019). To express the heteromeric hP2X2/3 receptor, cRNAs encoding His-hP2X2R and wt-hP2X3R were coinjected at a ratio (w/w) of 1:6. Oocytes were stored at 19°C in an oocyte ringer solution (ORi^+^) containing 90 mM NaCl, 1 mM KCl, 1 mM CaCl_2_, 1 mM MgCl_2_, and 10 mM HEPES (Carl Roth, Karlsruhe, Germany) adjusted to pH 7.4 with NaOH and supplemented with 50 μg/ml gentamicin (AppliChem, Darmstadt, Germany). The procedures followed for the maintenance of and the surgical treatment for *X. laevis* adults were approved by the governmental animal care and use committee of the State Agency for Nature, Environment, and Consumer Protection (LANUV, Recklinghausen, Germany; reference no. 81-02.04.2019. A355), in compliance with Directive 2010/63/EU of the European Parliament and of the Council on the protection of animals used for scientific purpose.

### 2.3 Two-electrode voltage clamp electrophysiology

Ion currents mediated by P2X receptors were evoked by the indicated concentration of ATP or α,β-meATP and were recorded 1 or 2 days after cRNA injection at ambient temperature at a holding potential of −60 mV by two-electrode voltage clamp (TEVC) as previously described (Hausmann et al., 2006). Calcium-free ORi^−^ solution (90 mM NaCl, 1 mM KCl, 2 mM MgCl_2_, 10 mM HEPES, pH 7.4) was used to avoid bias due to calcium-activated chloride channels (CaCC) endogenously expressed in *X. laevis* oocytes (Miledi, 1982; Methfessel et al., 1986). For recordings of wt-hP2X7R, the composition of the ORi^−^ solution was modified according to protocols described previously and contained 100 mM NaCl, 2.5 mM KCl, 1 mM MgCl_2_, 5 mM HEPES, pH 7.4 (Klapperstück et al., 2000). The oocytes were continuously superfused with ORi^−^ by gravity flow (5–10 ml/min). The agonists ATP or α,β-meATP and the investigated NSAIDs were diluted in ORi^−^ on the day of the recording. The following agonist concentrations were used for the different P2X subtypes: 1 μM ATP (hP2X1R mutant, hP2X3R, rP2X3R, S^15^V-hP2X3R, and S^15^V-rP2X3R); 10 μM ATP (hP2X2R and hP2X4R); 300 μM free ATP^4-^ (hP2X7R); and 1 μM α,β-meATP (hP2X2/3R). A peak current protocol (Supplementary Figure S1) was used to analyze fast- and intermediate desensitizing P2XR subtypes (P2X1R, P2X3R, and S^15^V-P2X3R), and a steady-state protocol (Supplementary Figure S2) was used for slowly or partially desensitizing P2X subtypes (P2X2R, P2X2/3R, and P2X4R). For recordings of P2X7R, a modified steady-state protocol was used (Hausmann et al., 2006). The application of different bath solutions was controlled by computer-operated magnetic valves controlled by the CellWorks E 5.5.1 software (npi electronic, Tamm, Germany).

### 2.4 Pig dorsal root ganglia preparation

The dorsal root ganglia (DRG) of pigs were sampled according to the 3R criteria for reductions in animal use, as leftovers from previous independent animal studies (e.g., LANUV reference no. 81-02.04.2018. A051). For this purpose, pigs of the German Landrace breed, with an average age of 15 weeks (14.6 SD2.7) and weight of 47.3 kg (SD11.2), were euthanized using an overdose of pentobarbital 60 mg/kg body weight. Subsequently, the DRG were collected. The DRG of pigs were transferred on ice, and fine excision was performed in ice-cold DMEM F12 medium containing 10% FBS and treated with 1 mg/ml collagenase P, 1 mg/ml trypsin T1426, and 0.1 mg/ml DNAse for digestion. Then, the DRG were cut into small pieces inside the digestion medium for surface enlargement and incubated at 37°C and 5% CO_2_ for 120 ±30 min. After approximately 60 min in the digestion medium, the DRG were triturated using a glass pipette. After the full incubation time, they were triturated thrice using custom-pulled glass pipettes with decreasing tip diameters (from ∼1.1–1.2 to ∼0.3–0.4 mm). For further purification, the DRG were centrifuged at 500 G and 4°C twice for 4 minutes each, and the pellets were suspended in DMEM F12 with 10% FBS. They were subsequently separated from lighter cell fragments and myelin by centrifugation of a Percoll gradient containing a 60% Percoll and a 25% Percoll gradient for 20 min at 500 G. DRG neurons were plated on coverslips coated with poly-D-lysine (100 μg/ml), laminin (10 μg/ml), and fibronectin (10 μg/ml). Neurons were then cultured in neurobasal A medium supplemented with B27, penicillin, streptomycin, and L-glutamine and used for voltage-clamp recordings after 12–72 h in culture.

### 2.5 Whole-cell patch-clamp recordings of pig DRG neurons

Whole-cell voltage-clamp recordings of DRG neurons were performed using glass electrodes with micropipette tip resistances of 1.3–3.5 MΩ, pulled and fire-polished with a Zeitz DMZ-puller. The intracellular solution contained 10 mM NaCl, 140 mM CsF, 10 mM HEPES, 1 mM EGTA, 5 mM glucose, and 5 mM TEA-Cl (adjusted to pH 7.3 using CsOH). The extracellular bathing solution contained 140 mM NaCl, 3 mM KCl, 1 mM MgCl_2_, 1 mM CaCl_2_, 10 mM HEPES, and 20 mM glucose (adjusted to pH 7.4 using NaOH). The liquid junction potential was corrected by −7.8 mV. Membrane currents were measured at room temperature, with a holding potential of −77.8 mV, using a HEKA EPC-10 USB amplifier. α, β-methylene ATP (10 μM) and 100 μM diclofenac were applied using a gravity-driven perfusion system during the recordings. PatchMaster/FitMaster software (HEKA Electronics) and IGOR Pro (WaveMetrics) were used for data acquisition and analysis. Signals were digitized at a sampling rate of 5 kHz. The low-pass filter frequency was set to 10 kHz. Series resistance compensation was between 2.5 and 11.1 MΩ.

### 2.6 Data analysis

The recorded TEVC currents were analyzed using CellWorks Reader 6.2.2 (npi electronic, Tamm, Germany) and Microsoft Excel (Microsoft Corporation, Redmond, United States). The displayed current traces were generated using IGOR Pro 6.21 (WaveMetrics, Portland, United States) and edited with Microsoft PowerPoint (Microsoft Corporation, Redmond, United States). To generate concentration–response curves, non-linear regression analysis was performed using GraphPad Prism 5 (GraphPad Software, San Diego, United States).

Antagonist concentration–response data and IC_50_ values were calculated by normalizing ATP-induced responses to the control responses (recorded in the presence and absence of the antagonist, respectively). The four-parameter Hill equation (Eq. 1) was iteratively fitted to data collected from a minimum of four independent repeat experiments to obtain antagonist concentration–response curves and IC_50_ values.

$$\frac{I_{Ant}}{I_{\max}}=\frac{top-bottom}{1+{\left(\frac{\left[Ant\right]}{{IC}_{50}}\right)}^{nH}}+bottom$$ (1)

I_max_ is the control response in the absence of the antagonist, I_Ant_ is the response at the given antagonist concentration (Ant), and IC_50_ is the antagonist concentration that causes 50% inhibition of the response elicited by a given agonist concentration. The ratio between the response in the presence of a certain antagonist concentration and the control response in the absence of the antagonist is indicated as “% control current”.

In the case of fast-desensitizing P2XR subtypes (P2X1R and P2X3R), ATP is applied five times in repetition, and the ATP-induced current amplitude in the presence (after pre-incubation) of the antagonist (fourth application) is compared to the arithmetic mean of flanking control ATP-induced current amplitudes in the absence of the antagonist (third and fifth ATP application). Since some of the investigated NSAIDs showed an enduring, potentially irreversible inhibitory effect on the current amplitude, only the preceding (third) ATP-induced current amplitude was used to calculate the control current. The typical run-down of current amplitudes between consecutive, repetitive ATP applications was considered by applying a correction factor. The correction factor was calculated as the ratio of the fourth ATP-induced current amplitude to the third amplitude (Eq. 3) when the experiment was performed in the absence of the antagonist. When the experiment was performed in the presence of the antagonist, the ATP-induced current amplitude of the preceding current (third amplitude) was multiplied by this correction factor/quotient (Eq. 4) to obtain a control current corrected for the run-down effect. Since the magnitude of the run-down varies from day to day and batch to batch of oocytes, the correction factor was determined on each day of the experiments and was calculated as the arithmetic mean of several recordings (at least 3–5 and on average 5) on each day. Based on many years of experience with corresponding measurements, it was determined that the correction factor should be >0.4 and should scatter by a max of 10% around the mean value between the different measurements of a day for its calculation to include data from the experimental measurement day in the evaluation.

$$correctionfactor=\frac{\mathrm{ATP‐induced}\mathrm{current}\mathrm{amplitude}4\mathrm{in}\mathrm{absence}\mathrm{of}\mathrm{antagonist}}{\mathrm{ATP‐induced}\mathrm{current}\mathrm{amplitude}3\mathrm{in}\mathrm{absence}\mathrm{of}\mathrm{antagonist}}$$ (2)

$$\%controlcurrent=\frac{\mathrm{ATP‐induced}\mathrm{current}\mathrm{amplitude}4\mathrm{in}\mathrm{presence}\mathrm{of}\mathrm{antagonist}}{\mathrm{ATP‐induced}\mathrm{current}\mathrm{amplitude}3\mathrm{in}\mathrm{absence}\mathrm{of}\mathrm{antagonist}\times \mathrm{correction}\mathrm{factor}}$$ (3)

In the case of hP2X7R, the control current in the absence of the antagonist had to be extrapolated (Supplementary Figure S4 and Supplementary Figure S5) presuming a linear increase in permeability during continuous ATP application (North, 2002) since the permeability of the receptor is affected by the antagonist.

The IC_50_ values are displayed as geometric means with 95% confidence intervals (95% CI). All other values (including % control current or % inhibition) are presented as arithmetic means ± SEM, if not otherwise stated. Values were compared using the *t*-test or one-/two-way analysis of variance and multiple comparison tests as indicated. Statistical significance was set at *p* < .05.

### 2.7 hP2X3R X-ray structure-based molecular-dynamics simulations and evaluation of diclofenac binding

The human ionotropic cation-selective ATP receptor P2X3 was modeled based on its structure in the apo-closed state (PDBID: 5SVJ) (Mansoor et al., 2016) and embedded in a 1-palmitoyl-2-oleoyl-phosphatidylcholine (POPC) bilayer using the *g_membed* functionality (Wolf et al., 2010) in GROMACS. Modeller was used to build the single-point mutant L191A of the P2X3 apo-closed state to make the supposed binding cavity of diclofenac easily accessible to simulate the binding event within the µs-time-scale of the simulations (Fiser and Sali, 2003). The standard protonation state at neutral pH was assigned to all residues. The system was simulated using GROMACS (Abraham et al., 2015) version 2021, with an integration time-step of 2 fs.

A pressure of 1 bar was applied semi-isotropically with a Berendsen barostat (Berendsen et al., 1984) using a time constant of 5 ps. A temperature of 310 K was maintained with a velocity-rescaling thermostat (Bussi et al., 2007). Van der Waals interactions were calculated with the Lennard–Jones potential and a cutoff radius of 1.2 nm, with forces smoothly switched to 0 in the range of 1.0–1.2 nm with no dispersion correction. The protein was described by the CHARMM36m (Huang et al., 2017) force field, lipids by the CHARMM36 force field (Klauda et al., 2010), and water by the TIP3P model (Jorgensen et al., 1983).

Na^+^ and Cl^−^ were added to provide a bulk concentration of approximately 50 mM NaCl. The crystal structure of hP2X3R in complex with the AF-219 negative allosteric modulator (PDB-ID: 5YVE) was aligned with apo-state hP2X3 (PDB-ID: 5SVJ); one diclofenac molecule per protomer was then fitted onto the AF-219 molecule using PyMOL and placed into the apo-state structure. Seven independent systems, each of the wildtype P2X3 apo-closed state and L191A apo-closed state, were simulated for more than 200 ns each, preceded by equilibration for about 200 ns: first with restraints on all heavy atoms and lipids in the *z*-direction, second on all heavy atoms, and third on backbone atoms only. All trajectories that showed a stable binding of diclofenac were selected and clustered with the GROMACS tool *gmx cluster* using the GROMOS algorithm; the cut-off for RMSD differences in a cluster was set to 0.35 nm.

Initial force-field parameters for diclofenac were generated according to the CHARMM generalized force field (CGenFF) (Vanommeslaeghe et al., 2010; Vanommeslaeghe et al., 2012; Vanommeslaeghe and MacKerell, 2012; Yu et al., 2012), using the CHARMM-GUI webserver (https://charmm-gui.org/). The initial molecular geometry and charge assignments were further optimized using the force-field toolkit (ffTK) (Mayne et al., 2013) version 2.1 plugin for the visual molecular dynamics (VMD) version 1.9.4a57 analysis suite (Humphrey et al., 1996). The ffTK program provides a workflow of quantum-mechanical calculations using ORCA (Neese et al., 2020) 5.0.3, followed by Newtonian optimizations using the nanoscale molecular dynamics (NAMD) (Phillips et al., 2020) engine. An initial parameter file is generated in ffTK by analogy using the protein structure file (psf) and protein coordinate file generated by CHARMM-GUI. The initial molecular geometry was optimized using ORCA at the MP2/6-31G* level of theory. After geometry optimization had converged, atomic partial charges were approximated using ORCA by calculating water-interaction energies at the HF/6-31G* level of theory. Aliphatic and aromatic hydrogens were assigned partial charges of .09 and .115, respectively; only hydroxyl hydrogens were optimized. To account for the positive charge associated with the dipole created by halogens known as alpha-holes, a lone-pair particle (LP) was added automatically *via* CHARMM-GUI (Pang et al., 2020). New bonded parameters for diclofenac only contained two dihedral terms, which are consistent with the CHARMM36 force field, used for diclofenac simulations; dihedral bonds were not further optimized due to having a bond energy penalty of less than 50 (unitless penalties as provided by the CGenFF program). Detailed instructions for using the most updated ffTK with support for the open-source quantum chemistry package, ORCA, can be found at the ffTK website (https://www.ks.uiuc.edu/Research/vmd/plugins/fftk) and the updated tutorial (https://www.ks.uiuc.edu/∼mariano/fftk-tutorial.pdf).

## 3 Results

### 3.1 Validation of the inhibitory effect of various NSAIDs on P2X3R-mediated currents

**FIGURE 1:**
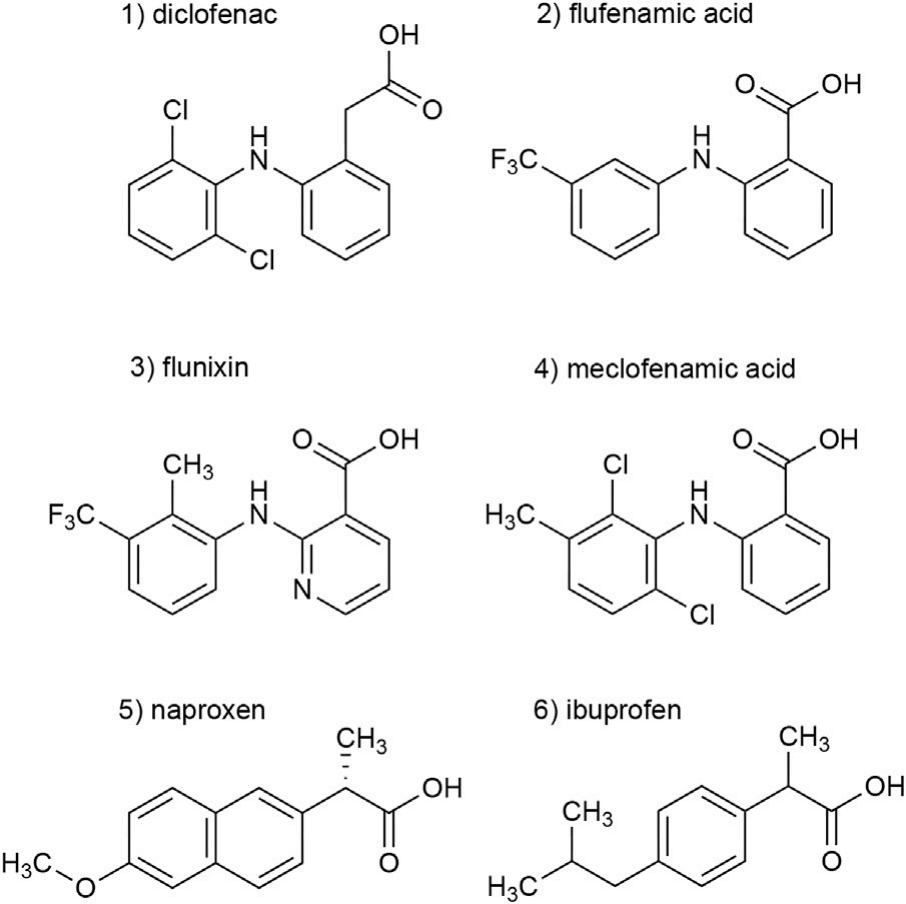
Chemical structures of the investigated NSAIDs (1–6).

In a previous screening of 2000 approved drugs, natural products, and bioactive substances, various NSAIDs were found to inhibit S^15^V-rP2X3R-mediated currents (Obrecht et al., 2019). These included diclofenac, flunixin meglumine, meclofenamic acid, and niflumic acid, where diclofenac showed the greatest inhibitory effect (>80% inhibition) of the screened NSAIDs (Obrecht et al., 2019). Since pharmacological analyses of P2X3Rs are hampered by fast desensitization, preventing a binding equilibrium being reached between ATP and a simultaneously present antagonist, we used the S^15^V-P2X3R mutant in the present study, which was shown to be more suitable for automated fluorescence-based screening and also facilitated the pharmacological characterization of specific P2X3R ligands by TEVC (Hausmann et al., 2014; Obrecht et al., 2019). To validate the screening results, we characterized the potency of diclofenac, flunixin, and meclofenamic acid using TEVC on *X. laevis* oocytes heterologously expressing S^15^V-rP2X3R and His-S^15^V-hP2X3R. Instead of niflumic acid, we decided to investigate the structurally related flufenamic acid (FFA). Furthermore, we included the NSAIDs ibuprofen and naproxen in our investigations because these are used extensively in daily practice. The structural formulas of the NSAIDs investigated are shown in Figure 1 (Figure 1, compounds 1–6).

**FIGURE 2:**
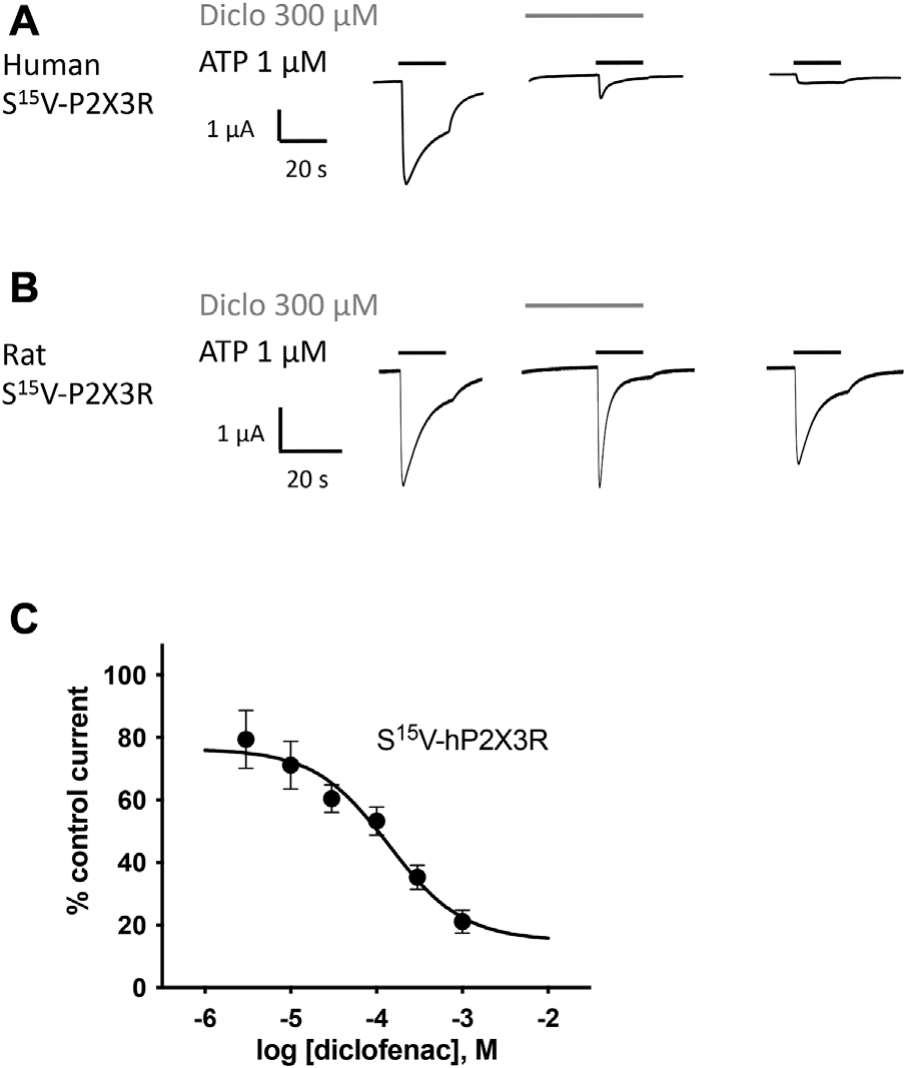
Diclofenac inhibition of ATP-induced currents of P2X3R is concentration-dependent. The effect of diclofenac on ATP-induced (1 μM) currents through S15V-hP2X3R **(A)** and S15V-rP2X3R **(B)** expressed in *X. laevis* oocytes was analyzed by TEVC electrophysiology. **(A,B)** Representative original current traces show the effects of 300 µM diclofenac on ATP-induced (black bars) currents of the indicated P2X3R. To ensure a binding equilibrium was reached, diclofenac was pre-incubated for 30 s before adding ATP (gray bar). **(C)** The resulting concentration–inhibition curve of diclofenac at hP2X3R exhibited an IC_50_ of 138.2 µM (95% CI: 46.0–415.1 µM). Data points represent the means and SEM.

A peak current protocol including a 30-s preincubation of the antagonist was applied (Supplementary Figure S1) to determine the inhibitory effect of the NSAIDs at P2X3Rs. The current amplitude in the presence of the antagonist was compared to that of the preceding control current in the absence of the antagonist. The inhibitory effect of diclofenac, FFA, and flunixin was not reversible by the following washout, which was reflected in a reduced amplitude of the subsequent control current that could not be explained by the run-down alone (c.f. Figure 2A). Due to this potentially irreversible inhibitory effect, the subsequent amplitude in the absence of the antagonist did not provide a suitable reference and was not taken into account to calculate the control current.

**FIGURE 3:**
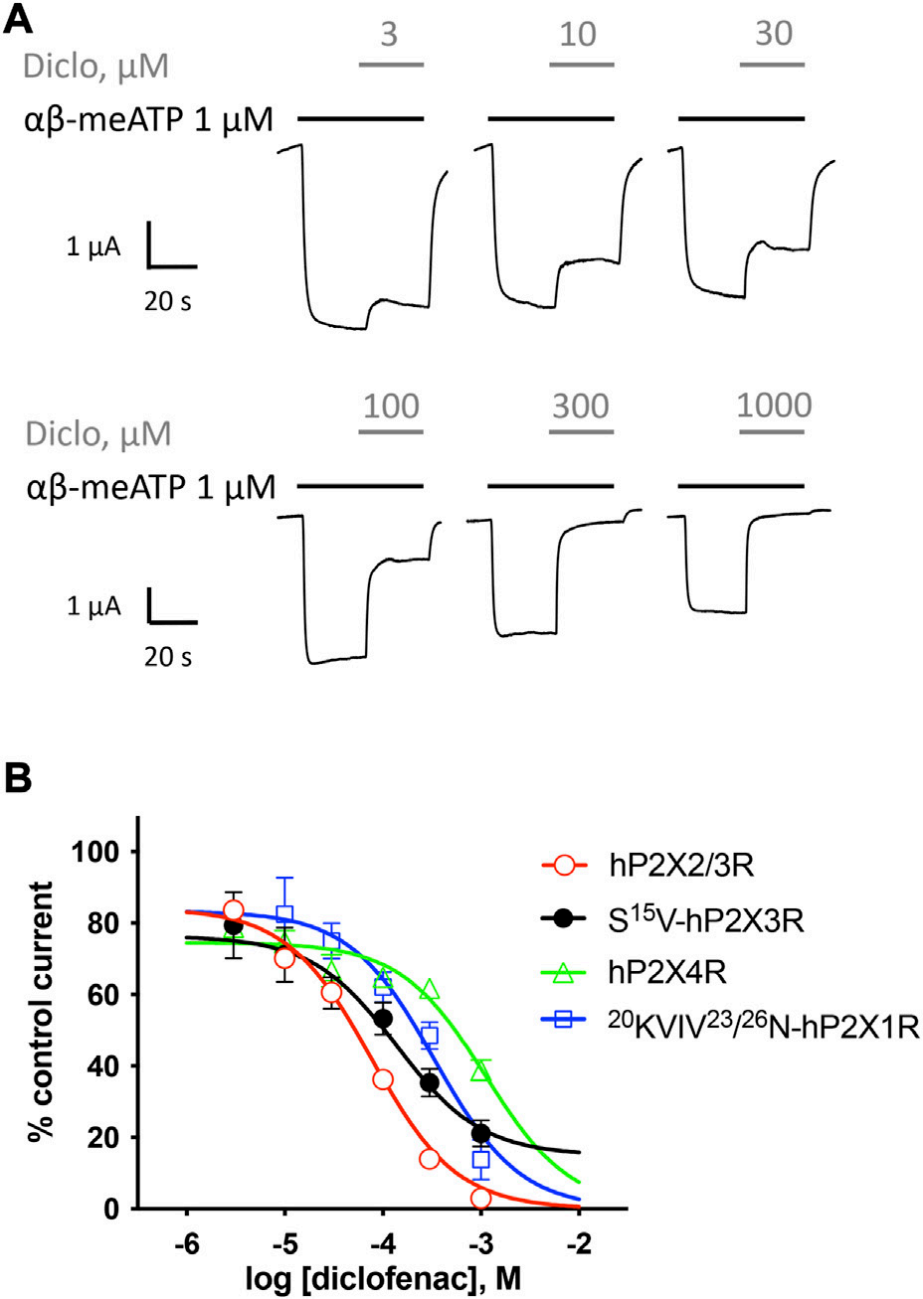
Effect of diclofenac at heteromeric hP2X2/3R and selectivity profiling. **(A)** Representative original current traces show the effects of 3–1000 µM diclofenac (gray bars) on α,β-meATP-induced (1 μM, black bars) currents through heteromeric hP2X2/3R expressed in *X. laevis* oocytes. **(B)** Concentration–inhibition curve of diclofenac at the indicated P2XR isoforms are shown as obtained from TEVC measurements using a peak current (P2X1R and P2X3R) or steady-state (P2X2/3R and P2X4R) protocol (c.f. Supplementary Figure S1/Supplementary Figure S2). All IC_50_ values are summarized in Table 2. Best-fit values of logIC_50_ values of hP2X2/3R and S15V-hP2X3R are significantly different (*p* = .0293). Best-fit values of logIC_50_ of ^20^KVIV^23^/^26^N-hP2X1R are significantly different from hP2X2/3R (*p* < .0001) but not from S15V-hP2X3R (*p* = .2409). Best-fit values of logIC_50_ of hP2X4R are significantly different from hP2X2/3R (*p* < .0001) but not from S15V-hP2X3R (*p* = .0917).

All investigated NSAIDs were less effective at the rat P2X3R than at the human P2X3R (Figures 2A, B) or even had no effect at all on rat P2X3R; thus, we decided to focus our further investigation on human P2X receptors. Concentration–response analysis revealed that ATP-evoked hP2X3R-mediated responses were inhibited by various NSAIDs. Desensitizing P2X receptors showed increased error bars with lower diclofenac concentrations (see for instance S^15^V-hP2X3 in Figure 2C/Figure 3B or the ^20^KVIV^23^/^26^N-hP2X1 in Figure 3B). This may be due to non-equilibrium during preincubation of low concentrations of diclofenac and/or the more error-prone evaluation of current amplitudes in the peak current protocol (Supplementary Figure S1) when using low diclofenac concentrations (likely due to the necessary estimation of the control current at the time of the combined agonist/antagonist based on the previous ATP application and the necessary correction factor consideration). Thus, we have refrained from testing concentrations below 3 μM because the increasing errors may have resulted in unreliable values. Diclofenac proved to be the most effective antagonist, with an IC_50_ value of 138.2 µM (95% CI: 46.0–415.1 µM; Figure 2C) and a maximum inhibition of ∼80% at a concentration of 1 mM (Table 1). FFA proved to inhibit hP2X3R-mediated currents with a non-significant lower potency (IC_50_ value of 221.7 µM; 95% CI: 98.9–496.8 µM; Table 1) in comparison to diclofenac. Flunixin, which is exclusively approved and used in veterinary medicine, had a significantly greater potency for the hP2X3 receptor than FFA (IC_50_ value of 32.4 µM; 95% CI: 11.6–90.2 µM; Table 1), but a maximum inhibition of only 53% at a concentration of 1 mM was observed, indicating a significantly lower efficacy of flunixin than of diclofenac. By contrast, only weak inhibition of hP2X3R was observed for ibuprofen, meclofenamic acid, and naproxen. The current amplitude in the presence of 100 μM meclofenamic acid, naproxen, or ibuprofen was reduced by a maximum of 15%–18%, suggesting an estimated IC_50_ value of >300 µM (Table 1). Due to their low inhibitory potency (ibuprofen, meclofenamic acid, and naproxen), low efficacy (flunixin) (c.f. Table 1), or their exclusive use in veterinary medicine (flunixin), these were not investigated further. Thus, only diclofenac—being the most effective antagonist—and FFA, due to its additional use in research, were further analyzed.

**TABLE 1:** Concentration–response analysis of NSAIDs at S15V-hP2X3R expressed in *X. laevis* oocytes. n.d., not determined. 1) Best-fit values of logIC_50_ values of diclofenac and flufenamic acid are not significantly different (*p* = .3071). 2) Best-fit values of logIC_50_ values of diclofenac and flunixin are not significantly different (*p* = .0775). 3) Best-fit values of logIC_50_ values of flufenamic acid and flunixin are significantly different (*p* = .001). 4) Mean values of diclofenac and flufenamic acid are not significantly different (*p* = .1813). 5) Mean values of diclofenac and flunixin are significantly different (*p* = .0017). 6) Mean values of flufenamic acid and flunixin are not significantly different (*p* = .0537).

	IC_50_, µM	95% CI IC_50_	Max. inhibition at 1 mM	n
Diclofenac	138.2^1,2^	46.0–415.1	78.9% ± 3.7%^4,5^	10
Flufenamic acid	221.7^1,3^	98.9–496.8	69.8% ± 3.8%^4,6^	10
Flunixin	32.4^2,3^	11.6–90.2	53.7% ± 4.8%^5,6^	8
Ibuprofen	>300	n.d	n.d	8
Meclofenamic acid	>300	n.d	n.d	9
Naproxen	>300	n.d	n.d	9

### 3.2 Characterization of the potency and selectivity of diclofenac at P2X receptors by TEVC

Selectivity profiling of diclofenac was performed at hP2X1R, hP2X2/3R, hP2X2R, hP2X4R, and hP2X7R (Figure 3B). To analyze the heteromeric hP2X2/3R, the ATP derivate α,β-meATP was used (Figure 3A) to evoke hP2X2/3R-mediated currents because it does not activate the homotrimeric hP2X2R. Currents mediated by the homotrimeric hP2X3R can be neglected due to its strong desensitization and run-down, when α,β-meATP is applied repetitively in short intervals (Bianchi et al., 1999; North, 2002). The desensitization kinetics of the heterotrimeric hP2X2/3 receptor resemble those of the homotrimeric hP2X2 receptor; therefore, the steady-state protocol was used (Figure 3A) (North, 2002). Diclofenac antagonized the heteromeric P2X2/3R with the highest potency of 76.7 µM (95% CI: 64.6–91.2 µM) and showed the following rank order of its potencies at P2XR subtypes: hP2X2/3R > hP2X3R > hP2X1R > hP2X4R. However, it should be noted that the differences in the IC_50_ values were not significantly different between hP2X3R, hP2X1R, and hP2X4R. All IC_50_ values are summarized in Table 2. By contrast, at the hP2X2R, diclofenac did not antagonize ATP-evoked P2X2R-mediated responses, but the presence of diclofenac exhibited a 1.7-fold increase in the P2X2R responses, and thus showed a potentiating effect at the P2X2R. Neither 100 µM diclofenac nor 300 µM FFA induced current at hP2X2R in the absence of ATP (Supplementary Figure 3 SA).

**TABLE 2:** Concentration–response analysis of diclofenac at the indicated hP2XR subtypes expressed in *X. laevis* oocytes. *, no inhibition, but potentiation (c.f. Supplementary Figure S3); n.d., not determined. 1) Best-fit values of logIC_50_ values of hP2X2/3R and S15V-hP2X3R are significantly different (*p* = .0293). 2) Best-fit value of logIC_50_ of^20^KVIV^23^/^26^N-hP2X1R is significantly different from hP2X2/3R (*p* < .0001). 3) Best-fit value of logIC_50_ of^20^KVIV^23^/^26^N-hP2X1R is not significantly different from that of S^15^V-hP2X3R (*p* = .2409). 4) Best-fit value of logIC_50_ of hP2X4R is significantly different from that of hP2X2/3R (*p* < .0001). 5) Best-fit value of logIC_50_ of hP2X4R is not significantly different from that of S15V-hP2X3R (*p* = .0917). 6) Mean values of hP2X2/3R and S^15^V-hP2X3R are significantly different (*p* < .0001). 7) Mean values of^20^KVIV^23^/^26^N-hP2X1R and hP2X2/3R are significantly different (*p* = .0376). 8) Mean values of^20^KVIV^23^/^26^N-hP2X1R and S^15^V-hP2X3R are not significantly different (*p* = .3381). 9) Mean value of hP2X4 is significantly different from all other determined values (*p* = < .0003).

	IC_50_, µM	95% CI IC_50_	Max. inhibition at 1 mM	n
^20^KVIV^23^/^26^N-hP2X1R	337.8^2,3^	88.7–643.1	86.2% ± 4.9%^7,8^	6
hP2X2R	no inh.*	no inh.*	no inh.*	10
hP2X2/3R	76.7^1,2,4^	64.6–91.2	97.0% ± 0.9%^6,7^	13
S15V-hP2X3R	138.2^1,3,5^	46.0–415.1	78.9% ± 3.7%^6,8^	10
hP2X4R	1,113^4,5^	208.2–5,948	61.2% ± 2.8%^9^	8
hP2X7R	>> 300	n.d	n.d	9

In summary, diclofenac was shown to be significantly more potent at hP2X2/3R (IC_50_ 76.7 µM; 95% CI: 64.6–91.2 µM) than at hP2X3R (IC_50_ 138.2 µM; 95% CI: 46.0–415.1 µM). However, it should be noted that the use of different agonists (α,β-meATP hP2X2/3R; ATP hP2X3R) complicates the assessment of a quantitative comparative analysis.

To assess the effect of diclofenac on the non-desensitizing hP2X7R, a modified steady-state protocol was used. For recordings of P2X7R, most scientists use divalent-free solutions such as ORi supplemented with 100 µM flufenamic acid (FFA) as an unselective ion channel blocker to inhibit non-specific chloride conductance in the absence of divalent ions (Weber et al., 1995; Hülsmann et al., 2003). However, since FFA is one of the investigated NSAIDs, this supplement was not reasonable. Therefore, the composition of the ORi-solution was modified as follows: 100 mM NaCl, 2.5 mM KCl, 1 mM MgCl_2_, 5 mM HEPES, pH 7.4, and according to former protocols (Klapperstück et al., 2000), a free ATP^4−^concentration of 300 µM was adjusted. About 300 µM of diclofenac reduced the ATP^4-^-induced current amplitude by approximately 33% (Supplementary Figure S4), which suggested an estimated IC_50_ value of >300 µM for diclofenac at hP2X7R. Since such high concentrations of diclofenac are clinically irrelevant, we refrained from performing a concentration–response analysis.

### 3.3 Mechanism of action of diclofenac

**FIGURE 4:**
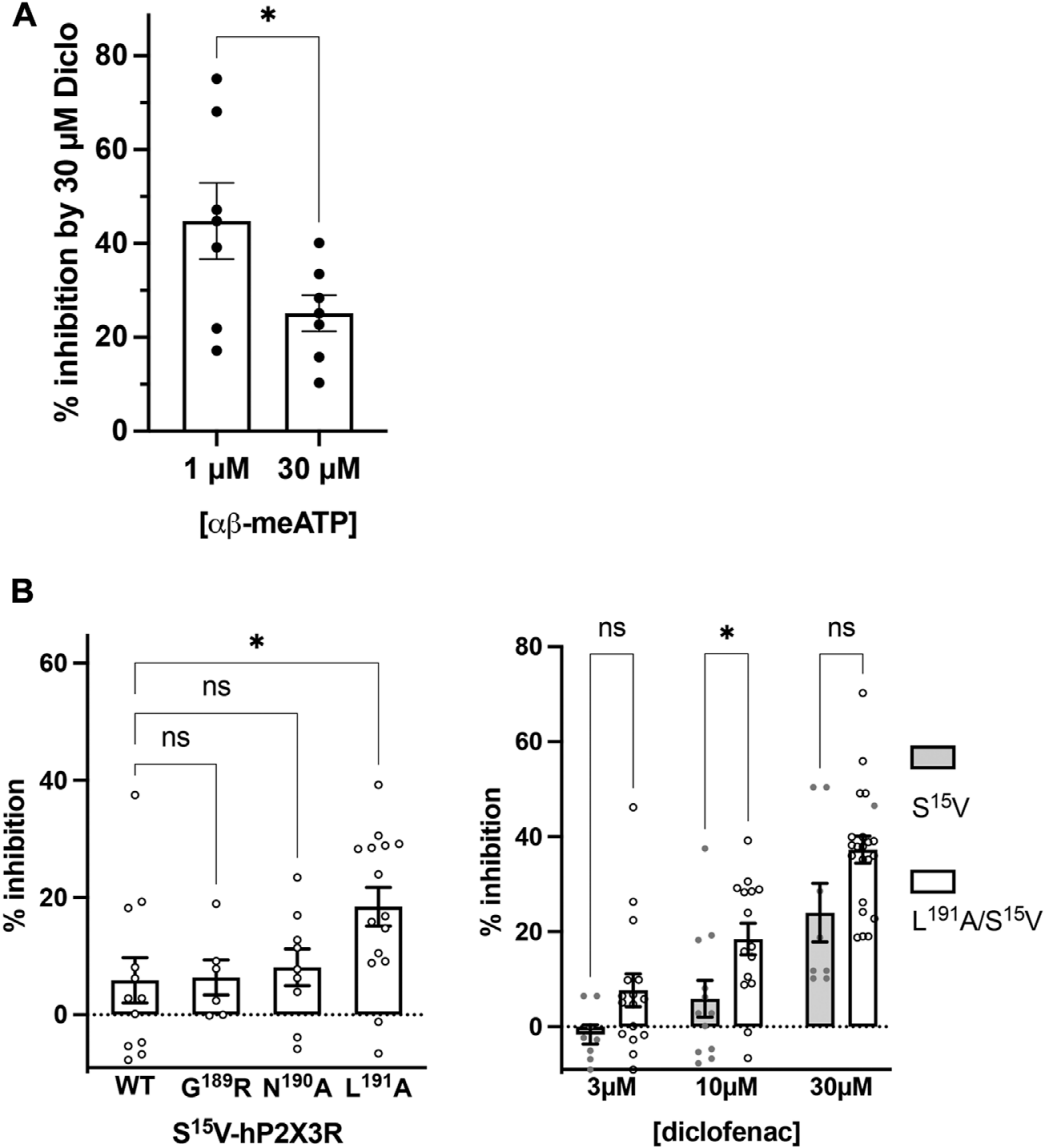
Diclofenac inhibition is modulated by the agonist concentration and the L^191^A mutant. **(A)** Bar graph showing the inhibition of the heteromeric hP2X2/3R by 30 µM diclofenac as determined with agonist concentrations of 1 µM or 30 µM α,β-meATP as indicated (% inhibition by 30 µM diclofenac ±SEM: 1 µM α,β-meATP, 44.8% ± 8.1%, *n* = 7; 30 µM α,β-meATP, 25.1% ± 3.8%, *n* = 7; *t*-test *p* = .049). **(B)** Left panel: comparative analysis of diclofenac inhibition of 10 µM ATP-induced currents of S^15^V-hP2X3R (“WT”), G^189^A/S^15^V-hP2X3R, N^190^A/S^15^V-hP2X3R, and L^191^A/S^15^V-hP2X3R. Bar graphs showing the inhibition of the indicated receptor by 10 µM diclofenac (% inhibition ±SEM: S^15^V (WT) −5.9% ± 3.9%, *n* = 12; G^189^A/S^15^V 6.4% ± 3.0%, *n* = 6; N^190^A/S^15^V 8.1% ± 3.1%, *n* = 9; L^191^A/S^15^V 18.4% ± 3.3%, *n* = 15; ANOVA multiple comparison showed that only the L^191^A/S^15^V was significantly different (*p* = .0243 (*) from S^15^V (WT), while G^189^A/S^15^V (*p* = .9996 (ns)) or N^190^A/S^15^V (*p* = .9536 (ns)) were not. Right panel: comparative analysis of diclofenac inhibition of 10 µM ATP-induced currents of S^15^V-hP2X3R and L^191^A/S^15^V-hP2X3R. Bar graphs showing the inhibition of the indicated receptor by 3, 10, or 30 µM diclofenac (% inhibition ±SEM: 3 µM diclofenac: S^15^V −1.6% ± 2.02%, *n* = 8 and L^191^A/S^15^V 7.7% ± 3.5%, *n* = 16, ANOVA multiple comparison *p* = .285 (ns); 10 µM diclofenac: S^15^V 5.9% ± 3.9%, *n* = 12 and L^191^A/S^15^V 18.4% ± 3.3%, *n* = 15, ANOVA multiple comparison *p* = .046 (*); 30 µM diclofenac: S^15^V 24.0% ± 6.2%, *n* = 8 and L^191^A/S^15^V 37.3% ± 2.8%, *n* = 20, ANOVA multiple comparison *p* = .053 (ns).

Although the S^15^V mutant of P2X3R desensitizes slowly (Hausmann et al., 2014), the desensitization may still prevent the reliable assessment of the mechanism of antagonism (Hausmann et al., 2014). Thus, the non-desensitizing heteromeric hP2X2/3R was used to assess the mechanism of action of diclofenac, which was also inhibited by diclofenac with the highest potency. To this end, the extent of inhibition of heteromeric hP2X2/3R by 30 µM diclofenac was determined using α,β-meATP concentrations of 1 or 30 µM as an agonist. We refrained from determining full agonist concentration–response curves at hP2X2/3R because simultaneous activation of homomeric hP2X2R occurs when hP2X2 and hP2X3 subunits are co-expressed and agonist concentration exceeds 30 µM α,β-meATP (Spelta et al., 2002). Approximately 30 μM diclofenac inhibited 1 or 30 µM α,β-meATP-induced current responses of the heteromeric hP2X2/3R by 44.8% ± 21.9% or 25.1% ± 10.1%, respectively (Figure 4A). Thus, inhibition by 30 µM diclofenac could be overcome by increasing concentrations of the agonist α,β-meATP, indicating competition of diclofenac and α,β-meATP at hP2X2/3R.

To further support the competitive nature of the inhibition and to exclude the possibility that diclofenac does bind in the negative allosteric site of hP2X3R as do other modulators (or negative allosteric antagonists) such as gefapixant (formerly AF-219) (Wang et al., 2018) or ATA (Obrecht et al., 2019), we examined mutations of amino acid residues in the allosteric site with respect to the effect of diclofenac. The allosteric site is formed by the lower body and dorsal fin domains of one subunit and the left flipper and lower body domains from the adjacent subunit and is thus located more closely to the transmembrane domains than the distinct ATP binding site (distance between the centers of the allosteric site and the ATP binding sites ∼15 Å; minimal distance between both sites ∼6 Å), which is formed by the head domain, upper body, and left flipper domains of one subunit and lower body and dorsal fin domains of the adjacent subunit (Mansoor et al., 2016; Wang et al., 2018). We have analyzed N^190^A and the G^189^R mutants [in the background of the S^15^V-hP2X3R (Obrecht et al., 2019)] of the negative allosteric binding site described in previous studies (Wang et al., 2018; Obrecht et al., 2019). These were inhibited by 10 µM diclofenac to a similar extent as the S^15^V-hP2X3R (Figure 4B), suggesting that the negative allosteric site of hP2X3R is not the binding site of diclofenac. In contrast to the findings for negative allosteric antagonists gefapixant/AF-219 (Wang et al., 2018) or ATA (Obrecht et al., 2019), the L^191^A/S^15^V-hP2X3R mutant was inhibited to a markedly greater extent by 10 µM diclofenac compared to the S^15^V-hP2X3R (Figure 4B). However, whether the greater inhibition of the L^191^A/S^15^V-hP2X3R mutant by diclofenac may be due to either an energetically more favorable diclofenac binding environment (see below) or a lower agonist potency of ATP remains unknown. A competitive mechanism of action is also compatible with our findings of diclofenac inhibition as well as FFA inhibition of the L^191^A/S^15^V-hP2X3R mutant shown in Supplementary Figure S6, which illustrates representative original current traces of the L^191^A/S^15^V-hP2X3R, showing the effects of 10 µM diclofenac or 30 µM FFA on ATP-induced currents. The initial inhibition by diclofenac or FFA at the beginning of the co-application could be overcome by prolonged ATP co-application (arrow), suggesting the competitive binding of ATP and the antagonist to the ATP-binding site. However, we did not quantify these findings because the time course of solution exchange may have varied between experiments, which may affect the accuracy and thus precise quantification.

**FIGURE 5:**
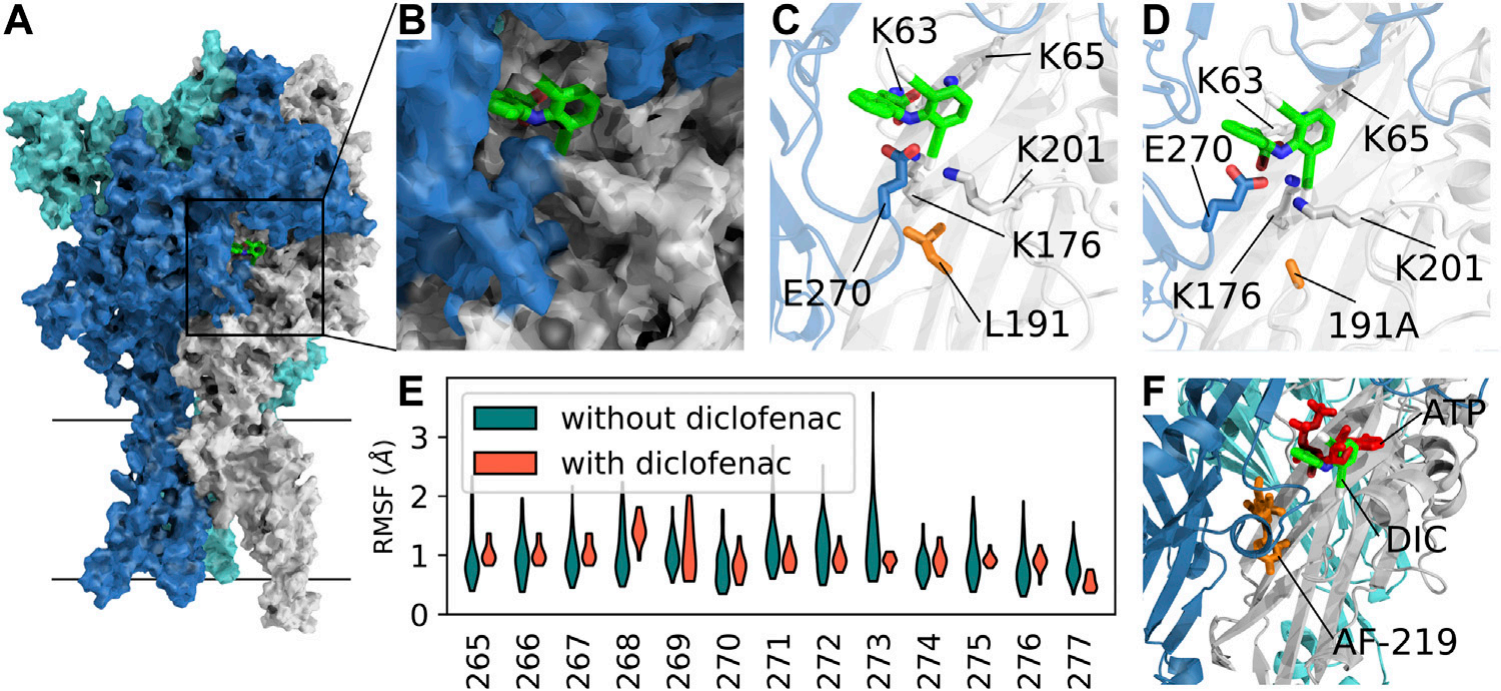
Competitive mechanism of action revealed by extensive all-atom molecular dynamics simulations. **(A)** Surface representation of apo-state P2X3 (PDB: 5SVJ) with bound diclofenac (obtained in our MD simulations) in the agonist-binding pocket between two adjacent subunits. **(B)** Close-up of the diclofenac-binding pocket as in **(A)**. **(C)** Average structure of the most frequently observed binding pose of diclofenac in P2X3 wildtype. Interacting residues within 4-Å distance are shown as sticks. **(D)** Same as C in the apo-state L191A mutant. The average structure of the first cluster of the independent simulations of the mutant shows a nearly identical binding pose. **(E)** RMSF (root-mean-squared fluctuation) of the left flipper domain for apo-state P2X3 wildtype with and without bound diclofenac to the agonist binding pocket. **(F)** Open-state P2X3R with bound ATP (PDB: 5SVK, ATP shown in red) with an overlay of AF-219 (shown in orange) and diclofenac (DIC, shown in green) shows the spatial position of the three molecules bound to hP2X3R. The position of AF-219 and diclofenac was obtained from an alignment of hP2X3R in complex with the AF-219-negative allosteric modulator (PDB-ID: 5YVE, AF-219 shown in orange) and the apo-state hP2X3R (PDB-ID: 5SVJ) with bound diclofenac and the ATP-bound open state PDB: 5SVK). An alignment of the open-state hP2X3R with bound ATP (5SVK) and hP2X3R in complex with the AF-219 negative allosteric modulator (5YVE) is shown in Supplementary Figure S8).

To investigate the binding mode of diclofenac and to shed light on a possible inhibition mechanism, we performed extensive all-atom molecular dynamics simulations of hP2X3R (Figure 5A) embedded in a lipid bilayer and surrounded by a physiological NaCl-based solution. Since the L^191^A mutation appears to facilitate diclofenac binding in our experiments, we initially assumed that diclofenac interacts with the receptor at a similar site like the allosteric inhibitor AF-219 (Wang et al., 2018), although L^191^A was shown to reduce binding of this compound. Therefore, we aligned the crystal structure of hP2X3R in complex with the negative allosteric modulator AF-219 (Wang et al., 2018) with the apo-state hP2X3, with one diclofenac molecule per protomer fitted onto the position of AF-219*.* We assessed the stability of the diclofenac-binding pose in our MD simulations and observed that diclofenac reoriented and moved toward the ATP-binding pocket on time scales of hundreds of nanoseconds.

In the seven independent simulation replicas, with three diclofenac molecules each, we observed 14 stable binding events for the hP2X3 wild-type and 14 for the L191A mutant, with some dissociation events (Supplementary Figure S7). We consistently observed diclofenac to alternatingly form salt–bridge interactions between its carboxyl groups and residues K^65^, K^63^, and K^176^. Furthermore, diclofenac binding was stabilized by a hydrogen bond with E^270^ and interactions between one of diclofenac’s chlorine atoms at K^176^ and K^201^ (Figures 5B, C). A similar diclofenac binding pose was observed in simulations of L^191^A-hP2X3R. We speculate that the removal of the bulky hydrophobic sidechain of L^191^ may facilitate diclofenac binding by creating an energetically more favorable environment (Figure 5D). We then calculated the root-mean-squared fluctuations of the loop of the left flipper domain (residue stretch 265–277, hP2X3 numbering) and observed a reduction in flexibility upon diclofenac binding. Thus, it appears that diclofenac binding may partially rigidify this region and may thereby impair allosteric communication between the ATP-binding site and the lower body and transmembrane domains (Figure 5E). Summarizing, our simulations imply the binding pose of diclofenac, which is nearby but distinct from the binding pose of the allosteric inhibitor AF-219 and partially overlaps with ATP, suggesting a partially competitive inhibition mechanism (Figure 5F).

### 3.4 Effect of diclofenac at native P2XRs in DRG neurons

**FIGURE 6:**
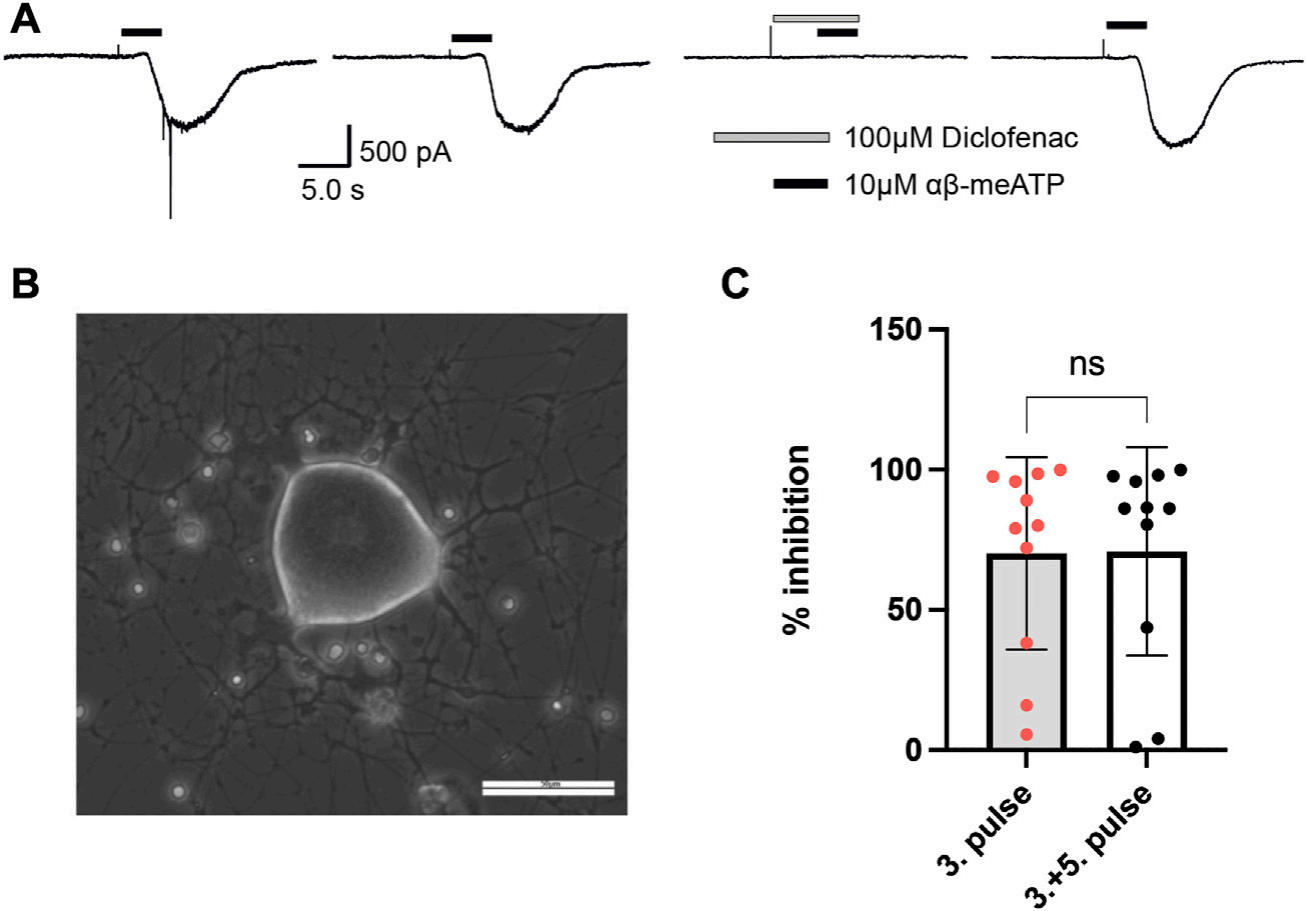
Diclofenac inhibition of P2X2/3R currents in dissociated porcine DRGs. **(A)** Representative original current traces of one porcine DRG neuron exposed four times for 3 s to 10 µM α,β-meATP in 3-min intervals. Please note that the neurons were exposed to five applications and that the first application is not shown here. Before the fourth application, 100 µM diclofenac was pre-incubated for 20 s before 10 µM α,β-meATP and 100 µM diclofenac were co-applied. **(B)** Representative picture of dissociated porcine DRGs at day 2 in culture. In the center a middle-sized DRG neuron is visible. Scale bar = 50 µm. **(C)** Bar graphs showing the relative diclofenac inhibition as calculated by the quotient of the max. α,β-meATP-induced peak current amplitude of the fourth application (in presence of diclofenac) vs. the preceding third ATP application (mean block ±SD = 70.2 ± 34.3%, *n* = 11) (left bar) or vs. the mean of the preceding (third) and following (fifth) α,β-meATP application [mean Block ±SD = 70.9 ± 37.2%, *n* = 11, *t*-test *p* = .963 (ns)] (right bar).

To examine whether diclofenac is capable of inhibiting native P2X3-subunit-containing receptors of nociceptive neurons with similar potency as oocyte-expressed recombinants hP2X2/3Rs and P2X3Rs, DRG neurons of pigs (3–4 months old) were analyzed. Currents elicited by 10 μM α,β-meATP were found in medium-sized (∼35–60 μm diameter) porcine DRG neurons. 10 μM α,β-meATP was applied repeatedly every 3 min for 3 s duration onto cultured porcine DRG neurons (Figures 6A, B). Whole-cell currents elicited by α,β-meATP appeared as a slowly activating and non-desensitizing phenotype mediated by heteromeric P2X2/3Rs. These were inhibited by 100 μM diclofenac (pre-equilibrated for 20 s) by 70.5% ± 35.8% (*n* = 11) (Figure 6C). Thus, diclofenac inhibited native pig P2X2/3Rs expressed in medium-sized DRG neurons to a similar extent to hP2X2/3Rs heterologously expressed in *X. laevis* oocytes (c.f. Figure 3).

### 3.5 Characterization of the potency and selectivity of FFA at selected P2X receptors

**FIGURE 7:**
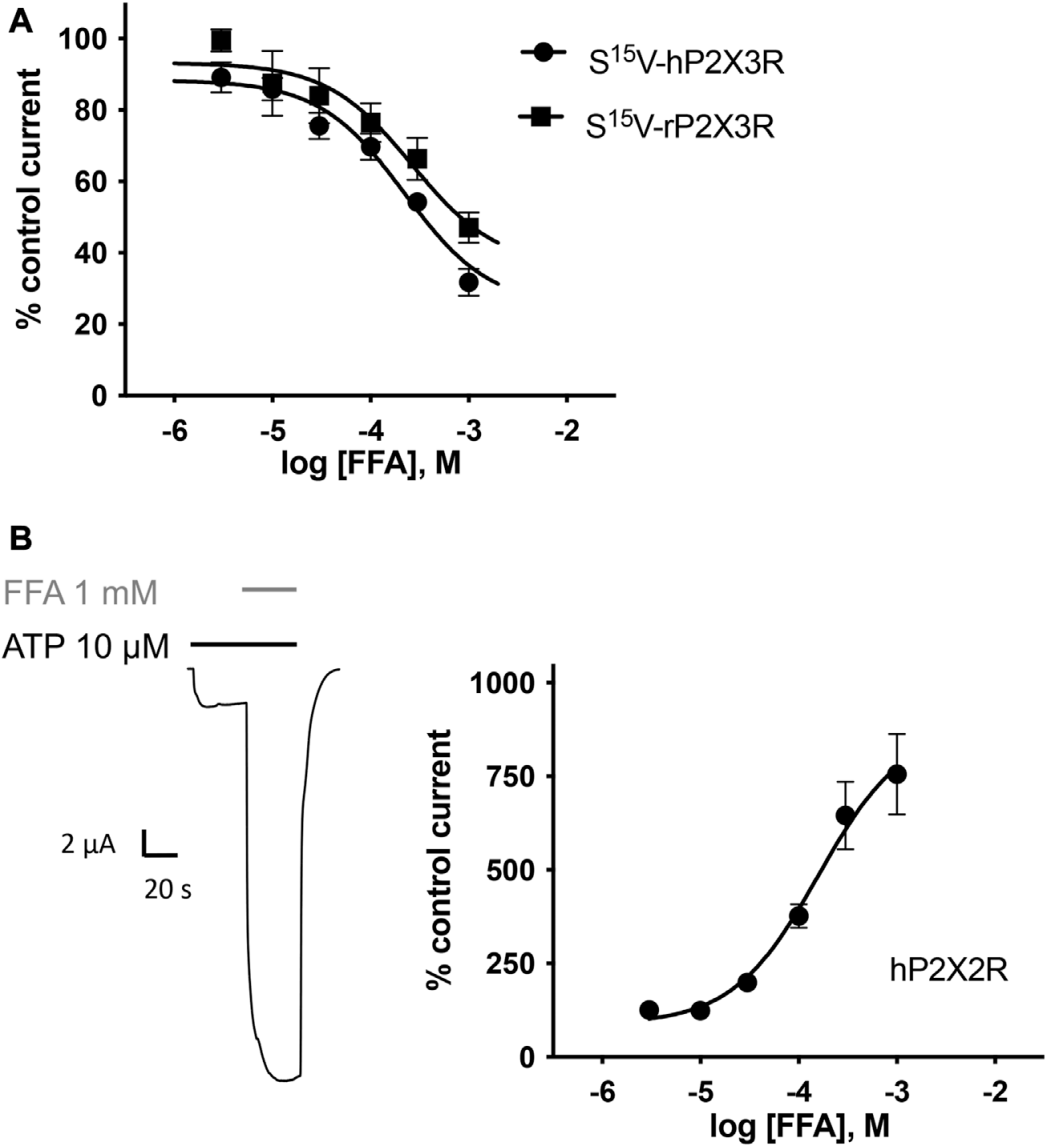
FFA inhibits P2X3R-mediated responses but potentiates hP2X2R-mediated responses. **(A)** Concentration–inhibition curve of FFA at human (●) and rat (▪) S^15^V-P2X3R exhibited IC_50_ values of 221.7 µM (95% CI: 98.9–497 µM) and 264.1 µM (95% CI: 56.9–612 µM), respectively. LogIC_50_ values are not significantly different (*p* = .197). Data points represent the means and SEM **(B)** Left panel: Representative original current trace shows the effect of 1 mM FFA (gray bar) on the ATP-induced (10 μM, black bar) current mediated by hP2X2R expressed in *X. laevis* oocytes. Right panel: Concentration–response curve of FFA at hP2X2R (●) exhibited a half-maximal potentiation value of 158.4 µM (95% CI: 64.4–389.7 µM; *n* = 8). Data points represent the means and SEM.

Concentration–response analysis revealed a concentration-dependent inhibition of hP2X3R- and rP2X3R-mediated currents by micromolar concentrations of FFA. IC_50_ values of 221.7 µM (95% CI: 98.9–497 µM) and 264.1 µM (95% CI: 56.9–612 µM) were determined at hP2X3R and rP2X3R, respectively (Figure 7A). In contrast to diclofenac, FFA did inhibit rP2X3R-mediated currents and was equipotent at hP2X3R and rP2X3R, (IC_50_ value of 221.7 and 264.1 μM, respectively, not significantly different; Figure 7A). This indicates a weaker selectivity of FFA toward the human P2X3R in comparison to diclofenac. Importantly, a concentration of 100 μM FFA exerted a relevant inhibitory effect of 30% on hP2X3R and 25% on rP2X3R (Figure 7A).

In the case of FFA, selectivity profiling was performed at hP2X2R and hP2X7R. These two subtypes were chosen because they either were potentiated or are often analyzed in the presence of FFA, respectively. When ATP and FFA were co-applied at the hP2X2R, the current amplitude increased up to 8-fold compared to the steady-state current in the absence of FFA (Figure 7B). Thus, in comparison to diclofenac, FFA shows a markedly higher potentiating effect on hP2X2Rs. The effect of FFA on hP2X7R-mediated currents was assessed by applying concentrations of 100, 300, and 1000 µM. A concentration of 100 μM exerted a relevant inhibitory effect of ∼39% (Supplementary Figure S5). A rough assessment of the IC_50_ value, using the three tested concentrations, suggested a value of approximately 900 μM for hP2X7R inhibition.

## 4 Discussion

### 4.1 Inhibition of hP2X3R-mediated currents as an additional mode of action of NSAIDs

Our findings demonstrate the inhibition of the human P2X3R by various NSAIDs. Diclofenac proved to be the most effective antagonist with an IC_50_ value of 138.2 µM. Among the investigated NSAIDs, diclofenac, FFA, and flunixin exerted an enduring, potentially irreversible inhibitory effect on the current amplitude, which could not be eliminated by the following washout period.

Considering the involvement of hP2X3R in nociception, it is conceivable that inhibition of hP2X3R contributes to the analgesic effect of NSAIDs and represents an additional mode of action besides COX inhibition. However, plasma levels and IC_50_ values must be taken into consideration. In the case of diclofenac, low nanomolar plasma levels are reached during the transdermal application, whereas 10–20-fold higher concentrations can be observed in synovial tissues (Efe et al., 2014). When injected intramuscularly, significantly higher plasma levels of approximately 1.8 μg/ml (∼6 μM) can be achieved (Drago et al., 2017). Similarly, maximum plasma levels of approximately 2.3–2.6 μg/ml (∼7–8 μM) can be achieved with the oral application of 50–75 mg diclofenac (Kurowski et al., 1994; Kienzler et al., 2010). According to our experimental data, the current amplitude of hP2X2/3R and hP2X3R was reduced by approximately 20%–30% in the presence of 3–10 µM diclofenac. Therefore, it can be assumed that clinically relevant concentrations of diclofenac exert a significant inhibitory effect on hP2X3R-mediated currents. However, diclofenac shows a many-fold higher potency at COX1 (IC_50_ value of .075 µM) and COX2 (IC_50_ value of .038 µM) (Warner et al., 1999) than at hP2X3R (IC_50_ value of 138.2 µM). The effect of diclofenac besides COX inhibition has also been studied by other groups, such as Gan (2010). For instance, in addition to COX inhibition, other effects such as the inhibition of acid-sensing ion channels (ASICs) were discovered (Voilley et al., 2001). However, the inhibition of P2X3R has not yet been described.

Similar to our findings for diclofenac, Hautaniemi et al. (2012) described the inhibition of P2X3R by the NSAID naproxen. According to our TEVC data, high micromolar to low millimolar concentrations of naproxen are necessary to inhibit hP2X3R, indicating a low potency of naproxen. These results are consistent with the results that Hautaniemi et al. (2012) obtained from calcium imaging of rat trigeminal neurons. Despite its lower potency in comparison to diclofenac, naproxen might as well exert a relevant inhibitory effect due to higher plasma levels. When administered orally, maximum plasma levels of approximately 70–80 μg/ml (∼300–350 µM) are reached after about 2 h (Desager et al., 1976; Dresse et al., 1978).

### 4.2 Selectivity profiling of diclofenac at P2X receptors and related side effects

Selectivity profiling of diclofenac at different P2XR subtypes showed strong inhibition of hP2X3R and hP2X2/3R and weaker inhibition of hP2X1R, hP2X4R, and hP2X7R. The rank order of its potencies is as follows: hP2X2/3R > hP2X3R > hP2X1R > hP2X4R > hP2X7R.

Diclofenac had a similar maximum inhibitory effect and potency at hP2X1R and hP2X3R. In contrast to its potentially irreversible effect on hP2X3R, diclofenac seemed to exert a reversible inhibitory effect on hP2X1R. Inhibition of hP2X1R, which is involved in inflammatory responses (Lecut et al., 2009), might contribute to the anti-inflammatory effect of diclofenac. However, the difference in potency between hP2X1R (IC_50_ 337.8 μM) and COX (IC_50_ value of 0.075 µM or 0.038 µM of COX1 or COX2, respectively) should be kept in mind.

Considering its low potency at hP2X4R and hP2X7R, it is unlikely that inhibition of these P2X subtypes, which are involved in nociception (Chessell et al., 2005; Tsuda et al., 2009), contributes to the analgesic effect of diclofenac.

Remarkably, diclofenac had a weak potentiating effect on hP2X2R-mediated currents. Regarding its strong inhibitory effect on hP2X3R and its weak potentiating effect on hP2X2R, a predominantly inhibitory effect on the heterotrimeric hP2X2/3 receptor could have been expected. Surprisingly, diclofenac proved to be more potent at hP2X2/3R (IC_50_ 76.7 µM) than at hP2X3R (IC_50_ 138.2 µM). However, it should be kept in mind that the use of the correction factor in hP2X3R measurements and the use of different agonists at hP2X2/3 and hP2X3R imply a bias that may affect an accurate, direct quantitative comparison. Since the run-down varies between different recordings, the inhibitory effect of diclofenac on hP2X3R might be underestimated.

It is presumed that taste disorders, which have been observed in clinical trials of newly developed P2X3R antagonists, mainly result from the inhibition of heterotrimeric P2X2/3R (Garceau and Chauret, 2019; McGarvey et al., 2022). Therefore, the question arises whether diclofenac might cause taste disorders due to its inhibitory effect on hP2X2/3R. While taste disorders as a side effect are listed as “very rare” in the prescribing information, more than 110 suspected cases of ageusia, dysgeusia, or taste disorders have been reported in the European Union so far in EudraVigilance (up to 28/11/2022) due to diclofenac administration (http://www.adrreports.eu/de/). It must also be assumed that there are a high number of unreported cases. In a prospective, randomized clinical trial regarding the postoperative administration of diclofenac, 58% of patients reported an impaired taste sensation (Attri et al., 2015). Therefore, it seems likely that diclofenac affects taste sensation due to the inhibition of hP2X2/3R.

### 4.3 Mechanisms of action of diclofenac at P2X3R and functional implications for gating

We have found the following lines of experimental evidence for competitive inhibition of the P2X3-subunit containing receptors by diclofenac: 1) inhibition by 30 µM diclofenac of hP2X2/3R could be overcome by increasing concentrations of the agonist α,β-meATP; 2) at L^191^A/S^15^V-hP2X3R, the inhibition by diclofenac or FFA at the beginning of the co-application with ATP could be overcome by prolonged ATP co-application; 3) the N^190^A and the G^189^R mutants of the negative allosteric binding site (Wang et al., 2018; Obrecht et al., 2019), which markedly affected the extent of inhibition of hP2X3R by the allosteric antagonists gefapixant/AF-219 or ATA, were inhibited by 100 µM diclofenac to a similar extent to hP2X3R.

In addition, our extensive all-atom molecular dynamics simulations have shown that the most common binding pose of diclofenac at hP2X3R largely overlaps with ATP bound to the open-state conformation of hP2X3R. Furthermore, we show by RMSF analysis that diclofenac when bound to hP2X3R alters the conformational flexibility of the left flipper and dorsal fin domains, crucially implicated in the ATP-induced gating of hP2X3R (Mansoor et al., 2016; Mansoor, 2022). Our simulation results also offer a mechanistic explanation for the inhibition of the ATP-induced gating of hP2X3R; the strong interactions of diclofenac with the residues K201 and E270 of the dorsal fin and left flipper domains, respectively, are likely to prevent the conformational rearrangements of the dorsal fin and left flipper domains. These are otherwise essential for channel gating or, mechanistically, for the transmission of ATP binding to the conformational rearrangement of the lower body domain, and eventually the opening of the ion channel pore.

According to our MD simulations, the strongest interactions of diclofenac and amino acid residues of the putative binding site were ionic interactions with lysines K63, K65, and K176 and hydrogen bonds with charged residues E270, K176, and K201. All of these amino acid residues are fully conserved between P2X subtypes and are essential for ATP binding, thus preventing targeted functional experiments for validating the diclofenac-binding pose. Therefore, we speculate that only non-conserved amino acid residues, dispensable for ATP binding or species-specific allosteric effects, may account for the differences in diclofenac-mediated effects between human and rat P2X3R. In summary, although awaiting further experimental confirmation, our results suggest that diclofenac acts *via* a similar mechanism of action to TNP-ATP (Mansoor et al., 2016).

### 4.4 Selectivity profiling of FFA at P2XRs questions its use in P2XR assays

FFA proved to inhibit human P2X3R-mediated currents with a similar potency (IC_50_ value of 221.7 µM) to diclofenac (IC_50_ 138.2 µM). In contrast to diclofenac, FFA did inhibit rat P2X3R-mediated currents (IC_50_ value of 264,1 µM), indicating a weaker selectivity of FFA toward the human P2X3R. As FFA is usually applied transdermally, plasma levels do not exceed 180 nM even with repetitive application (Drago et al., 2017). Due to its low potency at hP2X3R and its low plasma levels, it must be assumed that P2X3R inhibition does not contribute to the analgesic effect of FFA in a relevant manner.

However, the inhibitory effect of FFA on P2X3R could be of importance for other scientists who perform functional recordings of P2XRs with solutions supplemented with FFA. Being a non-selective ion channel blocker, FFA is widely used in research to avoid bias resulting from the activation of various other ion channels (Guinamard et al., 2013). Our findings demonstrate that a commonly used concentration of 100 μM FFA exerts a relevant inhibitory effect of approximately 30% on hP2X3R and 25% on rP2X3R. However, especially for recordings of P2X7R, FFA is often used as a supplement to divalent-free ORi-solution (Hülsmann et al., 2003). While inhibition of the P2X3R by FFA has not yet been described by other groups, there are contradictory results regarding its effect on P2X7R. Suadicani et al. (2006) suggested a competitive antagonism of FFA at P2X7R, whereas Ma et al. (2009) did not find an inhibitory effect of FFA on P2X7R, but rather an inhibition of Pannexin-1 by FFA. According to our data, hP2X7R-mediated currents are reduced by approximately 40% in the presence of 100 μM FFA. Even when FFA is no longer applied, the permeability of the receptor remains affected as shown in Supplementary Figure S5 by comparing the slope of the linearly increasing current before and after FFA application, which may indicate a potentially irreversible effect of FFA on hP2X7R-mediated current responses.

Taken together, our results question the use of FFA as a non-selective ion channel blocker when P2XR-mediated currents are to be measured and add another target to the already known unspecific ion-channel modulation by FFA (Guinamard et al., 2013).

In comparison to diclofenac, FFA shows a significantly higher potentiating effect on hP2X2R with a 7–8-fold increase in current amplitudes. This potentiating effect of FFA on hP2X2R has already been described by other groups (Schmidt et al., 2018). However, Schmidt et al. (2018) did not attribute this effect to a direct interaction of FFA with the receptor, but to membrane alterations caused by the amphiphilic FFA. However, from our point of view, this theory seems questionable since it cannot explain the opposing effects of FFA on hP2X2R and hP2X3R.

## 5 Conclusion/summary

In a previous screening of 2000 approved drugs, natural products, and bioactive substances, various NSAIDs were found to inhibit S15V-rP2X3R-mediated currents (Obrecht et al., 2019). Using TEVC, we identified diclofenac as a hP2X3R and hP2X2/3R antagonist with micromolar potency (with IC_50_ values of 138.2 and 76.72 µM, respectively), which was also shown to be effective in antagonizing native P2X2/3R-mediated responses in pig DRG neurons. Considering their involvement in nociception, the inhibition of hP2X3R and hP2X2/3R by micromolar concentrations of diclofenac may contribute to the analgesic effect of diclofenac and represent an additional, although less potent, mode of action besides the well-known COX inhibition. Our results support the presence of a competitive antagonism through which diclofenac, by interacting with residues of the ATP-binding site, left flipper, and dorsal fin domains, inhibits the gating of P2X3R by conformational fixation of the left flipper and dorsal fin domains. FFA proved to inhibit hP2X3R, rP2X3R, and hP2X7R, calling into question its use as a non-selective ion channel blocker, when P2XR-mediated responses are under study.

## Data Availability

The raw data supporting the conclusion of this article will be made available by the authors, without undue reservation. The data underlying the quantitative tables and figures are deposited at https://doi.org/10.6084/m9.figshare.22004789.v1.
