# Corrigendum: Diclofenac and other non-steroidal anti-inflammatory drugs (NSAIDs) are competitive antagonists of the human P2X3 receptor

**DOI:** 10.3389/fphar.2023.1225522

**Published:** 2023-06-02

**Authors:** Laura Grohs, Linhan Cheng, Saskia Cönen, Bassam G. Haddad, Astrid Bülow, Idil Toklucu, Lisa Ernst, Jannis Körner, Günther Schmalzing, Angelika Lampert, Jan-Philipp Machtens, Ralf Hausmann

**Affiliations:** ^1^ Institute of Clinical Pharmacology, RWTH Aachen University, Aachen, Germany; ^2^ Department of Neurology, University Hospital, RWTH Aachen University, Aachen, Germany; ^3^ Molecular and Cellular Physiology (IBI-1), Institute of Biological Information Processing (IBI), Jülich, Germany; ^4^ Department of Plastic Surgery, Hand and Burn Surgery, University Hospital RWTH Aachen, Aachen, Germany; ^5^ Institute of Physiology (Neurophysiology), RWTH Aachen University, Aachen, Germany; ^6^ Institute for Laboratory Animal Science and Experimental Surgery, RWTH Aachen University, Aachen, Germany; ^7^ Department of Anesthesiology, University Hospital RWTH Aachen, Aachen, Germany

**Keywords:** P2X3 receptor, nociception, chronic pain, non-steroidal anti-inflammatory drugs (NSAIDs), competitive antagonist, drug screening

Incorrect Supplementary Material

In the published article, there was an error in Supplementary Material PDF file.

1.) In the Supplementary Material PDF file the heading is incorrect. Instead of “Allosteric” it should be “Competitive” as it is also stated in the title of the main article. The correct heading of the Supplementary Material PDF file is “Diclofenac and other non-steroidal anti-inflammatory drugs (NSAIDs) are competitive antagonists of the human P2X3 receptor.”

2.) One author’s name has changed from Astrid Obrecht to Astrid Bülow. In addition, one authors is missing in the heading of the Supplementary Material PDF file. The author list should be Laura Grohs^1,2^, Linhan Cheng^1^, Saskia Cönen^1,3^, Bassam G. Haddad^3^, Astrid Bülow^1,4^, Idil Toklucu^5^, Lisa Ernst^6^, Jannis Körner^5,7^, Günther Schmalzing^1^, Angelika Lampert^5^, Jan-Philipp Machtens^1,3^ and Ralf Hausmann^1*^

The correct material statement appears below.

Heading of Supplementary Material PDF file:

“Diclofenac and other non-steroidal anti-inflammatory drugs (NSAIDs) are competitive antagonists of the human P2X3 receptor.”

Laura Grohs^1,2^, Linhan Cheng^1^, Saskia Cönen^1,3^, Bassam G. Haddad^3^, Astrid Bülow^1,4^, Idil Toklucu^5^, Lisa Ernst^6^, Jannis Körner^5,7^, Günther Schmalzing^1^, Angelika Lampert^5^, Jan-Philipp Machtens^1,3^ and Ralf Hausmann^1*^

The authors apologize for this error and state that this does not change the scientific conclusions of the article in any way. The original article has been updated.

