# Supplementary material for: Diclofenac and other non-steroidal anti-inflammatory drugs (NSAIDs) are competitive antagonists of the human P2X3 receptor
Source: Front Pharmacol. 2023 Mar 16;14:1120360. doi: 10.3389/fphar.2023.1120360 (PMC10060569; doi:10.3389/fphar.2023.1120360)
Supplement: Supplementary file 1 [file DataSheet1.pdf]

Supplementary Material

Diclofenac and other Non-Steroidal Anti-Inflammatory Drugs (NSAIDs) are Competitive Antagonists of the human P2X3 Receptor

Laura Grohs, Linhan Cheng, Saskia Cönen, Bassam G Haddad, Astrid Bülow, Idil Toklucu, Lisa Ernst, Jannis Körner, Günther Schmalzing, Angelika Lampert, Jan-Philipp Machtens, Ralf Hausmann*

***Correspondence:** Corresponding Author: rhausmann@ukaachen.de

1 Supplementary Figures and Tables

1.1 Supplementary Figures

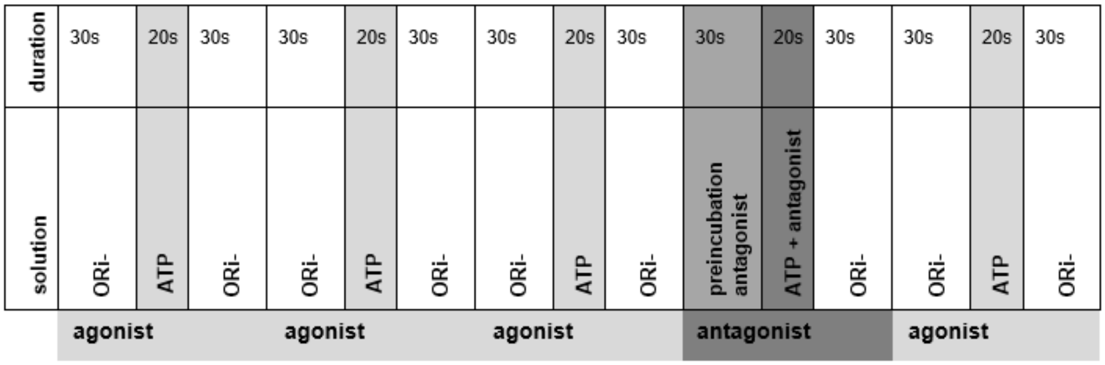

Supplementary Figure 1. Peak current protocol used for recordings of the desensitizing P2X1R and P2X3R mutants. First, the agonist ATP was applied three times for 20 seconds each to obtain a stable current amplitude as a reliable reference value. Each ATP application was followed by a wash out step with ORi- solution for 30 seconds. The wash out step was extended to 60 seconds to stabilize the baseline if necessary. After three applications of ATP and a washout step with ORi- solution, a 30-second preincubation with the antagonist and the simultaneous application of ATP and the antagonist for 20 seconds followed. Finally, ATP was once more administered in the absence of the antagonist.

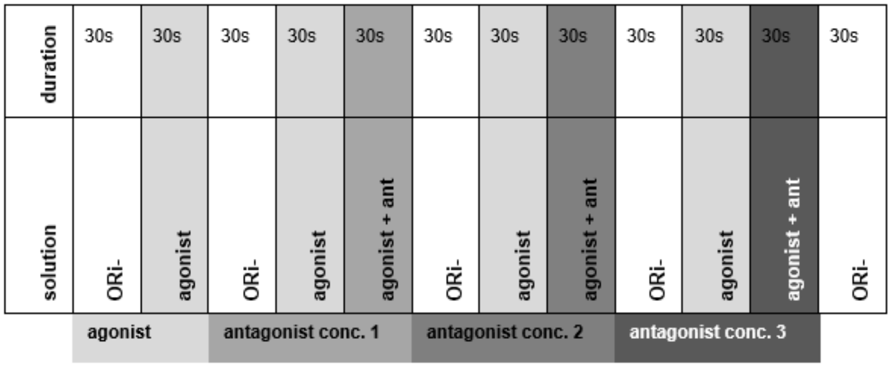

Supplementary Figure 2. Steady-state protocol used for recordings of the non- or partially desensitizing hP2X2R, hP2X2/3R or hP2X4R. In case of hP2X2R and hP2X4R the agonist ATP was applied to evoke current responses, whereas its derivate α,β-meATP was used for the heteromeric hP2X2/3R. When the agonist was applied, a steady state occurred after a few seconds, i.e. the current amplitude remained constant. After reaching the steady state, agonist and antagonist (ant) were co-applied. After a wash out step with Ori- this procedure could be repeated for further concentrations (conc.) of the antagonist. The wash out step with ORi- was extended to 40 seconds to stabilize the baseline if necessary.

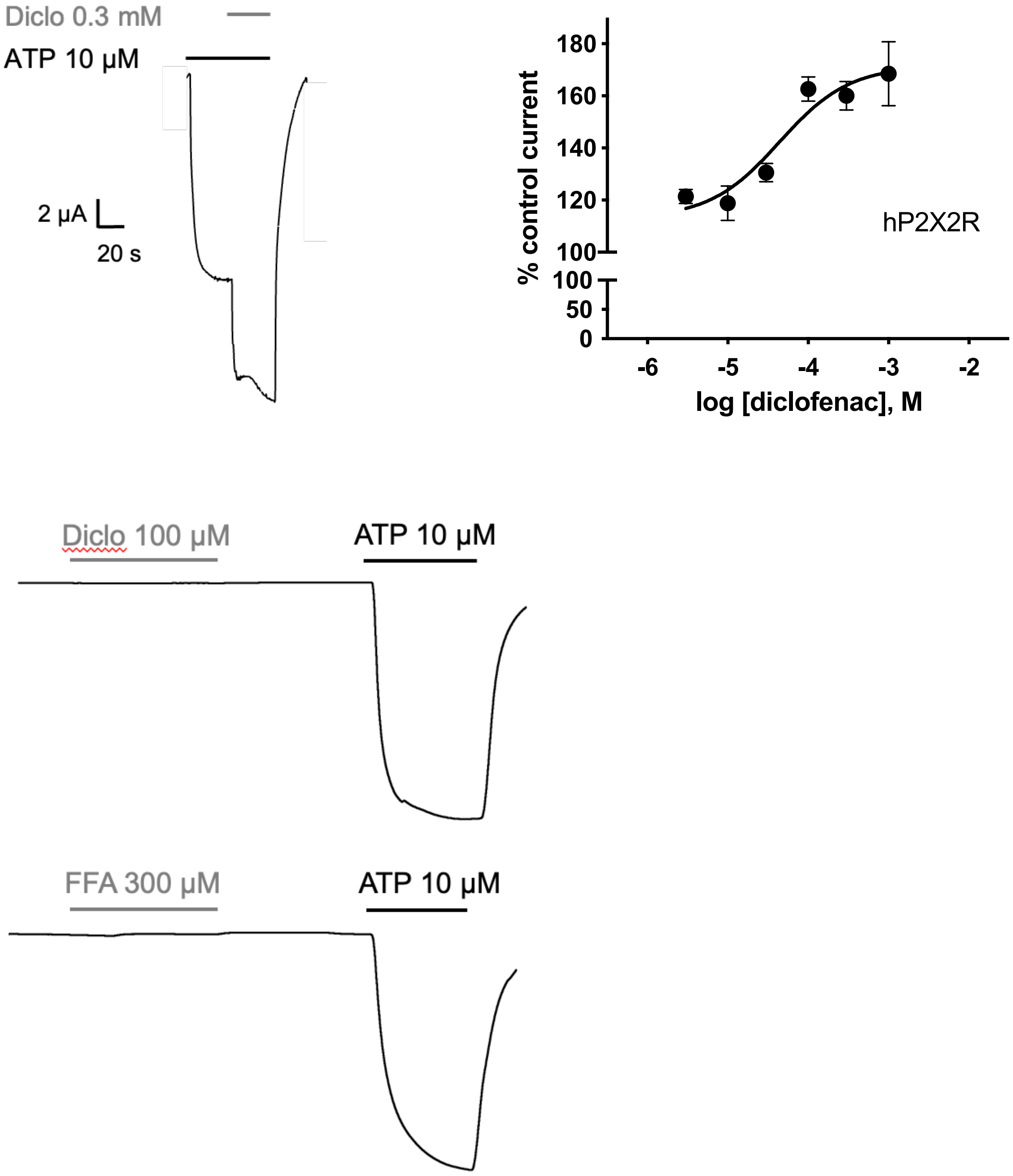

Supplementary Figure 3. Diclofenac potentiates hP2X2R-mediated responses. Upper left panel: Representative original current trace shows the effect of 300 µM diclofenac (grey bar) on the ATP-induced (10 µM, black bar) current mediated by the hP2X2R expressed in *X. laevis* oocytes. Upper right panel: Concentration–response curve of diclofenac at the hP2X2R (●) exhibited half maximal potentiation value of 158.4 µM (95% CI: 64.4 - 389.7 µM). Data points represent the means and SEM. Middle and lower panel: Representative original current traces showing that neither diclofenac nor FFA do exhibit agonist effects when applied in absence of ATP.

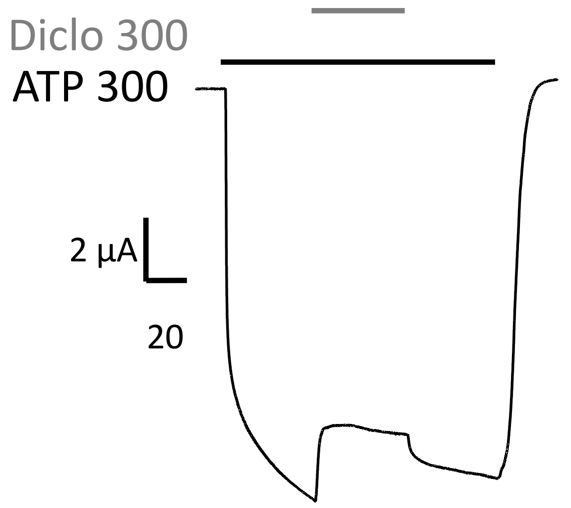

Supplementary Figure 4. Effect of diclofenac at hP2X7R. Representative original current trace shows the effects of 300 µM diclofenac (grey bars) on ATP^{4-}-induced (300 µM, black bar) currents through hP2X7R expressed in *X. laevis* oocytes.

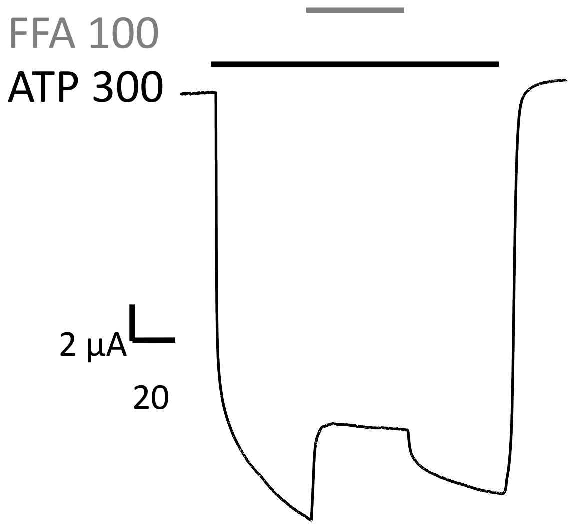

Supplementary Figure 5. Effect of FFA at hP2X7R. Representative original current trace shows the effects of 100 µM FFA (grey bars) on ATP^{4-}-induced (300 µM, black bar) currents through hP2X7R expressed in *X. laevis* oocytes.

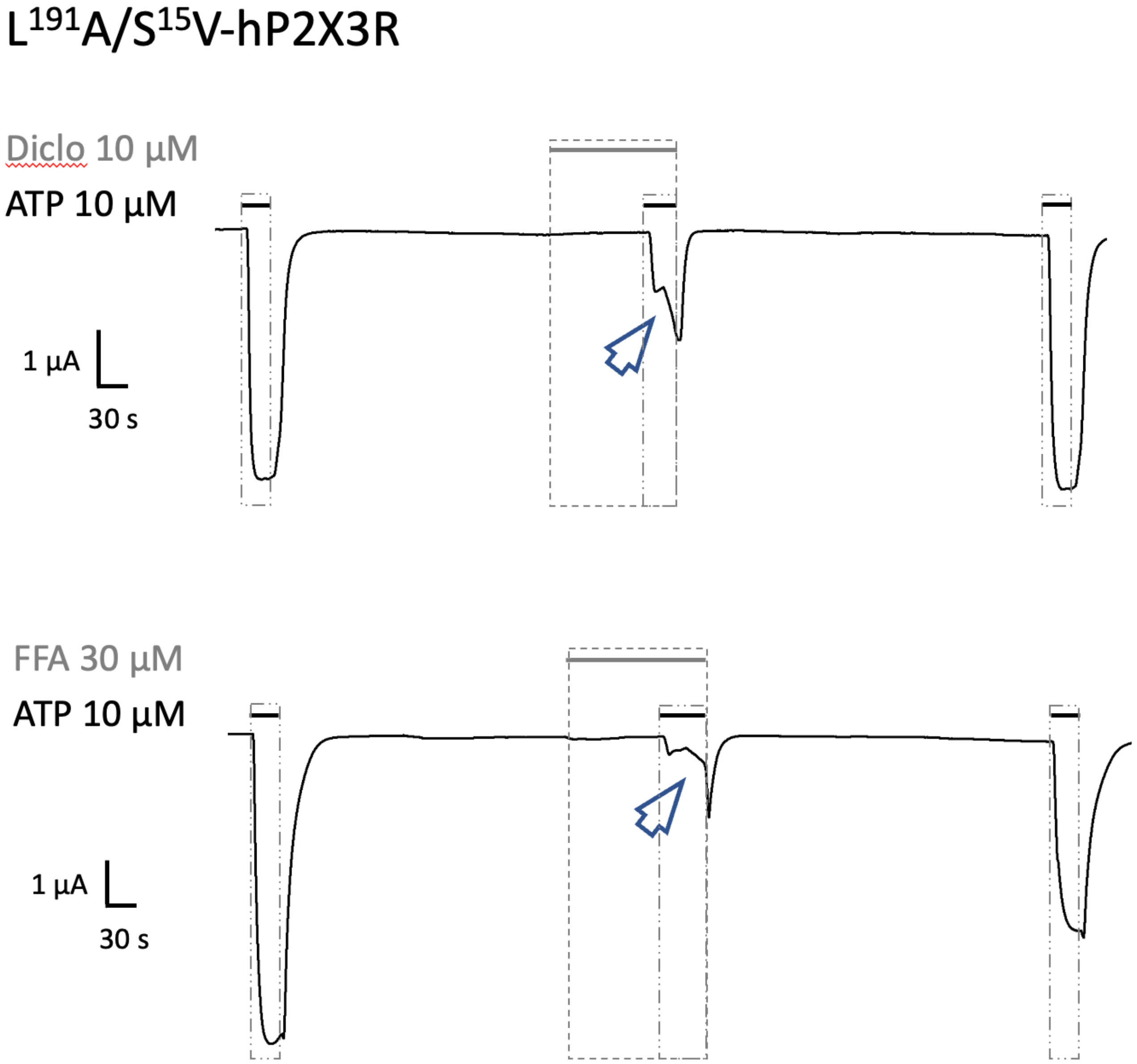

Suppl. Figure 6. Effect of diclofenac and FFA at the L^{191}A/S^{15}V-hP2X3R. Representative original current traces shows the effects of 10 µM diclofenac (upper panel, grey bar and dashed rectangular) or 30 µM FFA (lower panel, grey bars and dashed rectangular) on ATP-induced (10 µM, black bars and and dashed-dotted rectangular) currents (3rd, 4th and 5th ATP application of the peak current protocol are shown). Please note that the initial inhibition during co-application was overcome by prolonged ATP co-application (arrowheads).

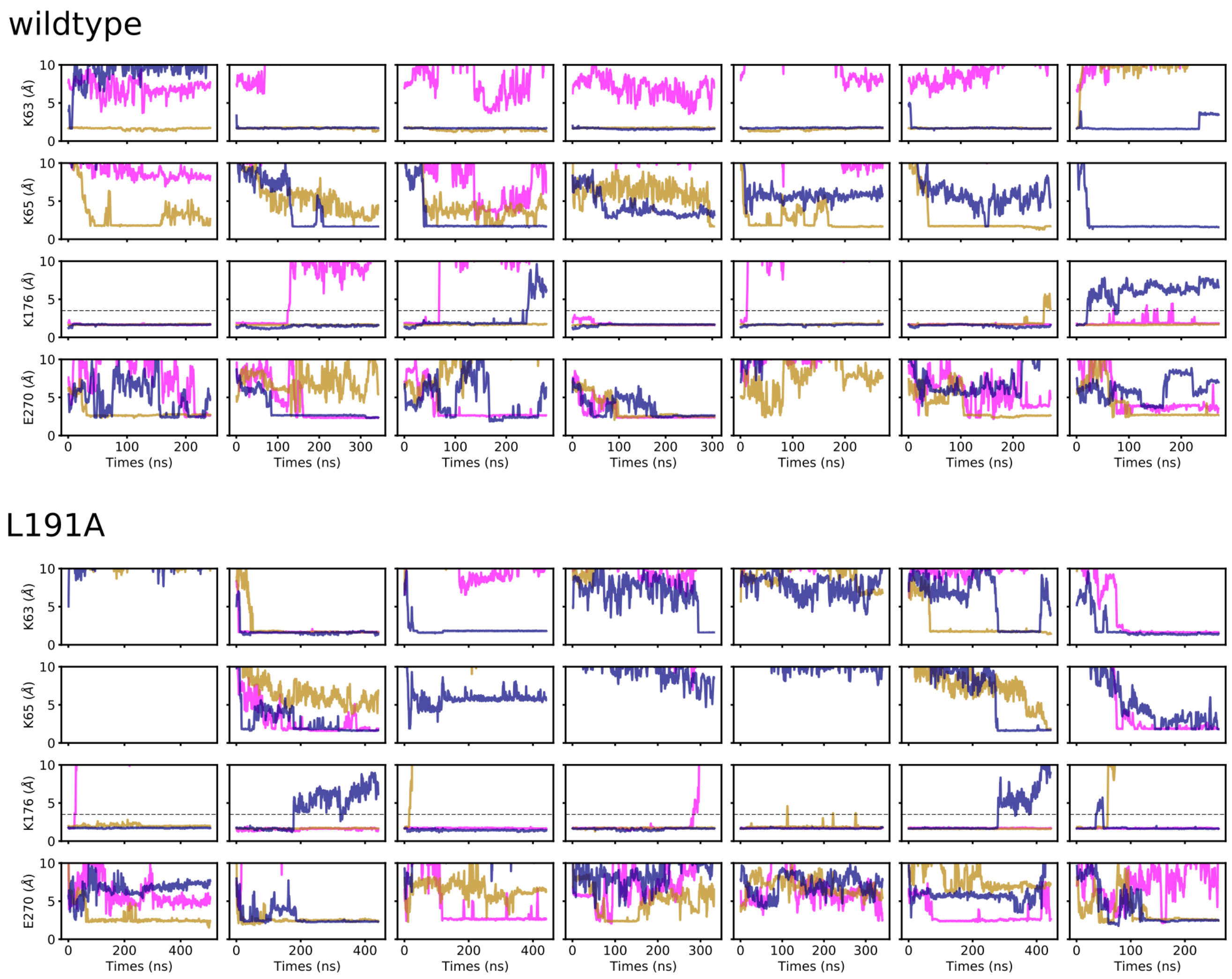

Suppl. Figure 7. Time course of minimal distances between diclofenac to K63, K65, K176 and E270 are shown for all trajectories over time. All trajectories, where the distance between diclofenac and K176 stayed stable below 3.5 Å (indicated by the dashed line for K176), were used for clustering for both P2X3 wildtype and L191A mutant.

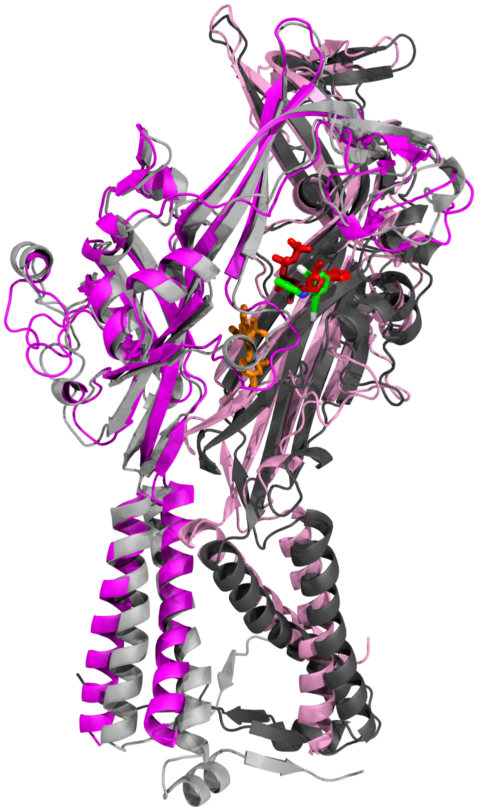

Suppl. Figure 8. Aligned open state hP2X3 with bound ATP (5SVK, shown in grey tones) and hP2X3 in complex with the AF-219 negative allosteric modulator (5YVE, shown in magenta tones). Only two adjacent protomers are shown for better visualization. Comparison of location of ATP (red), AF-219 (orange) and suggested binding pose for diclofenac (green) are shown as sticks (ATP in red, AF-219 in orange and diclofenac in green). The position of the AF-219 and diclofenac were obtained from an alignment of the hP2X3R in complex with the AF-219 negative allosteric modulator (PDB-ID: 5YVE, AF-219 shown in orange) and the apo state hP2X3R (PDB-ID: 5SVJ) with bound diclofenac and the ATP-bound open state PDB: 5SVK).

1.2 Supplementary Tables

Supplementary Table 1

Suppl. Table 1: Volumes and amounts of cRNA injected per oocyte for the different P2XR subtypes.

For the electrophysiologic recordings after 48 hours, a smaller amount of RNA was injected than for the recordings after 24 hours and the oocytes were stored overnight at 4°C instead of 19°C to achieve a similar expression on both recording days. To express the heteromeric hP2X2/3R, the cRNA of His-hP2X2R and wt-hP2X3R were coinjected. For the recordings of the P2X7 receptor, two different constructs (His-hP2X7 and wt-hP2X7) were tested.

**cRNA**	**Injected volume per oocyte [nl]**	**Injected amount of RNA per oocyte [ng]**
His-$S^{15}V$-hP2X3R	41	Recording after 24 hours: 3.7-4.9<br>Recording after 48 hours: 1.9-2.5
$S^{15}V$-rP2X3R	41	Recording after 24 hours: 3.7-3.9<br>Recording after 48 hours: 1.8-1.9
hP2X2/3R (co-injection of His-hP2X2$_A$R and hP2X3R)	41	Recording after 24 hours: 2.2 (His-hP2X2$_A$R) + 13.9 (hP2X3R)<br>Recording after 48 hours: 0.9 (His-hP2X2$_A$R) + 7.0 (hP2X3R)
His-hP2X2$_A$R	23	Recording after 48 hours: 0.1
His-hP2X4R	41	Recording after 48 hours: 48.0
His-^{20}RMVL^{23}KVIV^{23},S^{26}N-hP2X1R	23	Recording after 48 hours: 4.1
His-hP2X7R	41	Recording after 24 hours: 1.0
wt-hP2X7R	41	Recording after 24 hours: 4.8
